# The Effectiveness of Individualized Acupuncture Protocols in the Treatment of Gulf War Illness: A Pragmatic Randomized Clinical Trial

**DOI:** 10.1371/journal.pone.0149161

**Published:** 2016-03-31

**Authors:** Lisa Conboy, Travis Gerke, Kai-Yin Hsu, Meredith St John, Marc Goldstein, Rosa Schnyer

**Affiliations:** 1 New England School of Acupuncture, Newton, MA, United States of America; 2 Department of Epidemiology, College of Medicine and College of Public Health and Health Professions, University of Florida, Gainesville, FL, United States of America; 3 Boston Veterans Healthcare System/Jamaica Plain Campus, Boston, MA, United States of America; 4 University of Texas at Austin, Austin, TX, United States of America; Institute of Tropical Medicine (NEKKEN), Nagasaki University, JAPAN

## Abstract

**Background:**

Gulf War Illness is a Complex Medical Illness characterized by multiple symptoms, including fatigue, sleep and mood disturbances, cognitive dysfunction, and musculoskeletal pain affecting veterans of the first Gulf War. No standard of care treatment exists.

**Methods:**

This pragmatic Randomized Clinical Trial tested the effects of individualized acupuncture treatments offered in extant acupuncture practices in the community; practitioners had at least 5 years of experience plus additional training provided by the study. Veterans with diagnosed symptoms of Gulf War Illness were randomized to either six months of biweekly acupuncture treatments (group 1, n = 52) or 2 months of waitlist followed by weekly acupuncture treatments (group 2, n = 52). Measurements were taken at baseline, 2, 4 and 6 months. The primary outcome is the SF-36 physical component scale score (SF-36P) and the secondary outcome is the McGill Pain scale.

**Results:**

Of the 104 subjects who underwent randomization, 85 completed the protocol (82%). A clinically and statistically significant average improvement of 9.4 points (p = 0.03) in the SF-36P was observed for group 1 at month 6 compared to group 2, adjusting for baseline pain. The secondary outcome of McGill pain index produced similar results; at 6 months, group 1 was estimated to experience a reduction of approximately 3.6 points (p = 0.04) compared to group 2.

**Conclusions:**

Individualized acupuncture treatment of sufficient dose appears to offer significant relief of physical disability and pain for veterans with Gulf War Illness. This work was supported by the Office of the Assistant Secretary of Defense for Health Affairs through the Gulf War Illness Research Program under Award No. W81XWH-09-2-0064. Opinions, interpretations, conclusions and recommendations are those of the author and are not necessarily endorsed by the Department of Defense.

**Trial Registration:**

ClinicalTrials.gov NCT01305811

## Introduction

Clinical and registry programs indicate that 25% of the 700,000 veterans of the first Gulf War (Operation Desert Shield/Storm, years 1990–1991), are affected by clusters of symptoms and co-morbid medical diagnoses including chronic fatigue syndrome, fibromyalgia, irritable bowel syndrome, arthralgia, digestive complaints, and mood-related psychiatric disorders, including depression, posttraumatic stress disorder (PTSD), and other anxiety disorders [1,2]. Defined by the Centers for Disease Control and Prevention (CDC) [3], Gulf War Illness (GWI) is a complex, poorly understood illness, often with a highly individualistic presentation, and symptoms difficult for conventional medicine to treat effectively; GWI has been shown to be remarkably stable at 5- and 10- year follow-ups [1,4]. GWI is twice as prevalent in deployed veterans, and seen in 15% of non-deployed veterans [5]. There is no standard of care treatment for this syndrome at this time.

Although there are no published studies evaluating acupuncture’s effectiveness in the treatment of GWI, acupuncture has been shown to successfully reduce many key symptoms of GWI including pain [5,6], musculoskeletal disorders [7,8,9], both acute and chronic pain after amputation in military contexts [10,11], fatigue [12], state, trait and situational anxiety [13], and depression [14,15,16]. Further, there is evidence that acupuncture may be effective in the treatment of other complex diseases such as irritable bowel syndrome [17], fibromyalgia [18], and post-traumatic stress disorder [19] and that acupuncture is well tolerated by patients, safe, and may be cost-effective compared to routine care [20].

Chinese Medicine, on which acupuncture is based, uses diagnostic and treatment procedures that are complex [21] and tailored to each individual’s specific symptoms. Although this individualized treatment ideal is often replaced in clinical research with standardized protocols for the purposes of reliability and simplicity, the ability of Traditional Chinese Medicine (TCM) to be tailored to each patient is a core concept and strength that can be maintained successfully in a Randomized Controlled Trial (RCT) format [22]. This unblinded Phase II clinical trial utilized individualized treatment protocols, testing the effects of individualized acupuncture treatments offered in extant acupuncture practices.

## Materials and Methods

### Study design

Full trial design details have been published elsewhere [23]. Please see S1 File: Protocol. To maximize study compliance we employed acupuncturists to provide treatments in their own offices in communities where veterans work and live. We began recruitment of subjects and practitioners with a catchment area of 30 miles from our research study offices, and widened our catchment area with increasing study duration.

Veterans with Gulf War Illness were randomized to either (1) acupuncture treatment twice per week for 6 months or (2) the wait-list comparison group consisting of usual care from baseline for 2 months, followed by weekly treatments for 4 months. We chose an active control group to maximize internal validity while allowing us to gather preliminary data on minimal effective treatment dose. The two month wait time allowed us to judge if GWI symptom presentations are stable in our sample, as has been shown in other GWI samples [1,5]. Our treatment schedule duration, dose, and specific Chinese Medicine techniques employed are based on the clinical experience of our expert practitioners, and informed by literature review. Details of the protocol and implementation were determined before the trial began via focus groups with senior acupuncture faculty at the New England School of Acupuncture.

The New England Institutional Review Board approved this research protocol on September 4, 2009. All human participants gave written informed consent. All of our study processes were approved and oversight is provided by: 1) The New England Institutional Review Board (http://www.neirb.com/), 2) United States Army Human Research Protection Office (https://mrmc-www.army.mil/rodorphrpo.asp*)*. The study operated as planned between September 2009 and January 2013. Recruitment began immediately, and ran until July 2012. We initiated the clinical trial registration process before recruiting subjects, and registration was completed before we began to analyze the data. We confirm that all ongoing and related trials are registered. Please see S2 File. CONSORT Checklist.

#### Recruitment

We recruited via local advertisements and direct mailing to veterans of the first Gulf War drawn from the Defense Manpower Data Center (http://www.virec.research.va.gov/Non-VADataSources/DMDC.htm). Because the demographics of GWI veterans are unpublished, we did not know if there was a sufficient population near our study offices from which to draw our sample. Thus we designed the study to include treatment sites within a 100-mile radius of our study offices, and incorporated a mechanism to add treatment sites within that radius in areas where GWI veterans were found clustered. Thirty treatment sites were utilized. This design has the added benefit of allowing veterans to attain treatments near where they live and work, a technique that may have improved adherence.

#### Eligibility

**Fig 1:**
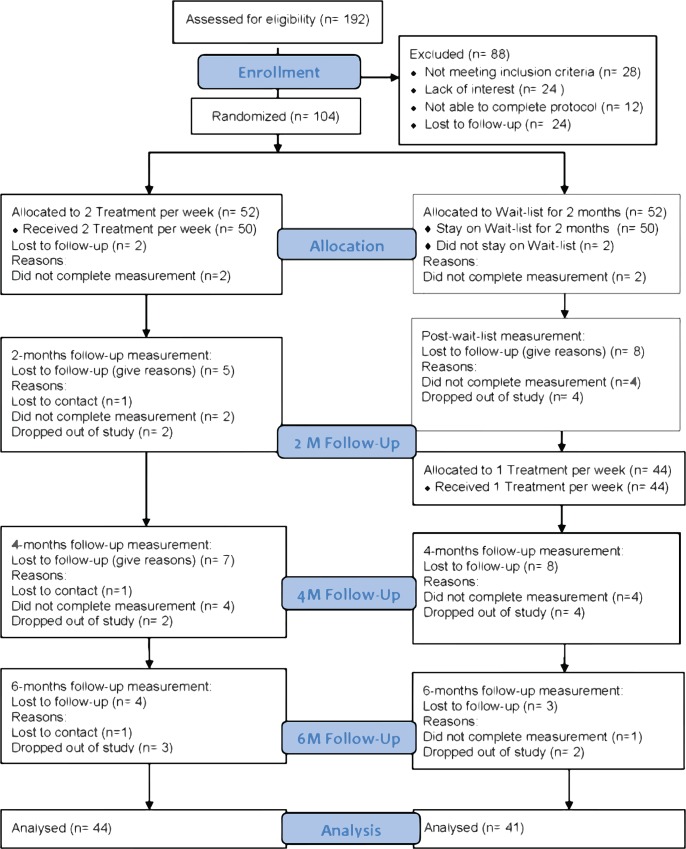
PLOS CONSORT Flow Diagram: Diagram of Screening, Randomization, and Follow-up.

All subjects met the illness definition of Gulf War Illness as determined by responses on the Gulf War Illness Symptom Checklist [5] and the inclusion/exclusion criteria set forth in the federal definition of Gulf War Illness as used for the Gulf War Registry (Please see Box 1). Subjects needed to pass through two eligibility screenings. First, a research assistant conducted a prescreening by phone, querying potential subjects about their illness and symptom experience. Second our study physician (MG) used the same criteria to complete an in-person medical screening. Our two-stage informed consent process included 1) verbal informed consent requested prior to the initial telephone screening, and 2) written informed consent administered in person, at the start of the screening visit. Please see Fig 1. An unblinded member of the study staff (LC) with no additional patient contact enrolled subjects. Study outcomes data were collected by electronic interface at our outpatient clinic. In fewer than 10% of cases, due to participants’ time constraints, participants were allowed to take their surveys home to fill out, and then mail back. During this screening visit, participants chose their future treatment practitioner from a list of practitioners convenient for them.

**Box 1: CDC Symptom Clusters for Gulf War Illness.**

Inclusion:

1. deployed to the “*Gulf Theater of operations*, *as defined by 38 CFR 3*.*317*, *includes Iraq*, *Kuwait*, *Saudi Arabia*, *Bahrain*, *Qatar*, *the United Arab Emirates*, *Oman*, *the Gulf of Aden*, *the Gulf of Oman*, *the Persian Gulf*, *the Arabian Sea*, *the Red Sea*, *and the airspace above all of these locations”* in the years 1990–1,
2. have at least 2 of the following symptoms from the 3 CDC clusters of symptoms that have lasted for more than 6 months. Each symptom cluster must be characterized as “mild-moderate” or “severe” with at least one symptom in each cluster required to be severe.
  A-Fatigability
    - fatigue 24 hours or more after exertion
  B-Mood and Cognition
    - feeling depressed or
    - feeling irritable or
    - difficulty thinking or concentrating or
    - feeling worried, tense, anxious or
    - problems finding words or
    - problems getting to sleep
  C-Musculoskeletal
  - joint pain or muscle pain

Exclusion:

Potential subjects will be excluded if they are:

- Currently enrolled in another clinical trial
- Have another disease that likely could account for the symptoms, as determined by our Medical Monitor
- Severe psychiatric illness (in the last 2 years psychiatric hospitalization, suicidal attempt, alcohol or substance abuse, use of antipsychotic medication) as measured by our primary screening instrument the Primary Care Evaluation of Mental Disorder (Prime MD).
- Unable to complete the protocol on based on the evaluation of the Medical Monitor
- Participants will not be excluded due to age, race, ethnicity, or gender limitations.
- Study is limited to United States veterans with Gulf War Syndrome, thus subjects could not be minors, illiterate, or unable to speak or understand English.

#### Group assignment and interventions

Collaborator RD randomly assigned participants to the two study arms using permuted block randomization with variable block sizes and assignments provided in sequentially numbered opaque sealed envelopes. After baseline evaluation and consent, a member of the study staff without involvement in data collection with subjects (LC) opened the assignment envelopes and recorded the assignment of each participant in a confidential log. LC then reported to the subject’s chosen practitioner the subject’s contact information and study assignment. The chosen practitioner then took responsibility of contacting the subject to schedule and begin treatments. LC also called and/or emailed the subject to report if acupuncture treatments were to begin immediately (group 1), or in 2 months (group 2).

Licensed acupuncturists with at least 5 years of clinical experience, who received additional in-house training concerning GWI, provided the acupuncture treatments. Although there are many styles of acupuncture within Chinese Medicine, acupuncturists were chosen who self-reported use of the TCM model of diagnosis. During the first session, the acupuncturist conducted an interview reviewing the subject’s medical history, symptoms and aspects of diagnosis from the perspective of TCM, including condition of the tongue, pulse, meridians, and acupoints. Each subject received an individualized diagnosis and treatment protocol addressing his or her unique pattern of symptoms. Brief interviews began each subsequent session, allowing patient and practitioner to prioritize symptoms, and identify any questions or concerns. Individualized treatment protocols allowed the practitioners to alter the treatment plan based on how the patient presented at the moment; including varying the selection of acupoints across treatments and adding particular co-interventions commonly used as part of TCM therapy to supplement manual needling (such as electroacupuncture for its efficacy in reducing pain and inflammation [24], heat therapies (e.g. heat lamp), Chinese massage, and press balls, tacks or magnets applied to points after needling). A sample treatment protocol is offered as S3 File.

Each session lasted approximately one hour. Acupoints were stimulated manually until “obtaining *de qi*,” a technique characteristic of TCM to elicit a response felt by both the patient and the acupuncturist. This needling sensation, adjusted for the comfort and safety of each patient, may be experienced as a pinch that rapidly subsides, or a sense of spreading pressure, dull ache, or warmth. Needles were retained for 30–45 min (10–35 stainless steel, disposable needles per session). After needle insertion, subjects were left to rest or nap. The type of needle, including gauge [32–38] and length (15–50 mm) as well as the depth of insertion (subcutaneous to about 25 mm) varied according to the area of the body being treated (i.e. extremities vs. trunk). Choice of acupoints could vary during subsequent treatments to improve results. Herbs and supplements were not allowed. Subjects were encouraged (but not required) to remain with the same acupuncturist for the whole study period to allow for development of patient-practitioner rapport.

#### Outcomes

A single measure of severity that addresses all possible presentations of GWI does not exist. Thus we chose to use the SF-36, a 36-item, well-validated and reliable general measure of health [25]. Given the importance of function on quality of life, we focused the main outcome to the SF-36 physical component scale score (SF-36P). Similarly, as pain is a common to most GWI presentations and very relevant in veteran health, our secondary outcome is the McGill Pain scale, a 15- item measure recording participants’ pain level and quality [26]. Outcomes were assessed with the assistance of a blinded study staff member at baseline, 2, 4, and 6 months. Raw data is offered as S4 File.

Subjects’ confidence in and usability of acupuncture were measured in a few ways. Participants were asked (1) their confidence in recommending acupuncture to a friend or family member using a five point scale from “Very Confident” to” Not Confident”, (2) the experience of the acupuncture using a five point scale from “Extremely Pleasant” to “Extremely Unpleasant”, (3) the experience of their relationship with their practitioner on a five point scale from “Extremely Pleasant” to “Extremely Unpleasant”, (4) how logical the acupuncture treatments were for them on a 6 point scale of “Very Logical” to “Not Logical at All”.

### Sample size

Our sample size was calculated to allow detection of clinically meaningful differences between treatment groups. Previous acupuncture research using our main outcome, the SF-36, in pain conditions [27,28] show a consistent standard deviation of 20 points in the SF-36 P for both baseline values and change scores. Sixty individuals per group (total n = 120) would offer us a power of 80% to detect the difference between groups of 7 points. Using Cohen’s d estimation of effect size [29] a sample size of 60 would allow us to see a moderate effect. In further support of our main outcome, a 7.8-unit improvement has been estimated clinically relevant for patients with similarly serious conditions [30,31]. We estimated a dropout rate of 10%.

### Statistical analyses

In our original proposal to the funder, to protect our main outcome from possibly large attrition, we proposed to initially test mean differences between groups following 2 months of treatment using Student’s t-tests at an alpha = 0.05. Using this strategy, we observed a mean reduction in SF-36 for Group 1 of 0.32 versus a reduction 4.53 for Group 2 (p = 0.22).

Of more interest to the study team is what changes might be seen after the clinically informed 6 month treatment window. This 6 month analysis is done by author TG and investigates whether those subjects assigned to biweekly acupuncture experienced differences in the SF-36P score over follow-up compared to those subjects who received weekly acupuncture following a 2-month delay. To assess potential differences, generalized estimating equation (GEE) models were fit in order to account for the correlation induced by repeated measurements on each subject. Under the assumption that baseline McGill pain is prognostic for SF-36P over time, model adjustment was made for baseline pain to increase precision in the estimated parameters.32 Eight subjects did not report this baseline measurement and, under the assumption that missingness was completely at random, these subjects were not included in the analysis set. In summary, we estimated the GEE model

$$SF36=\beta 0+\beta 1Itx+\beta 2It^{2}+\beta 3It^{4}+\;\beta 4It^{6}+\;\beta 5\rho \;+\;\beta 6ItxIt^{2}+\;\beta 7It^{4}+\;\beta 8ItxIt^{6}$$

where *Itx* denotes an indicator for biweekly acupuncture; *It*^*2*^,*It*^*4*^,*and It*^*6*^, are indicators for months 2, 4, and 6; *ρ* denotes baseline pain; and time was coded categorically to reflect suspected nonlinearities.

As a secondary analysis, the McGill pain score was similarly assessed for differences over time by treatment status. The time trend for the GEE fit was modeled categorically to account for nonlinear trends. The pain model was not adjusted for additional covariates, since no strongly prognostic variables were assumed to have been measured. All GEE models were fit using the software package geepack [33] in R version 3.1.1 under an exchangeable working correlation structure.

## Results and Discussion

### Study Population

Recruitment began in July of 2010 and the final follow-up visit was completed in January 2013. Please see Table 1 for baseline demographics and Fig 1. for study flow.

**Table 1:** Baseline Characteristics of the Study Population. Baseline Characteristics of the Study Population. Standard Deviations (SD) are offered for age, baseline pain, and baseline Sf-36(P).

Characteristic	Biweekly Treatment (N = 52)	Waitlist to Weekly Treatment (N = 52)
**Age-year +/- SD**	48.2 +/-9.9	48.2 +/- 3.5
**Female sex-N(%)**	7 (13%)	7 (13%)
**Self reported Race-N(% of total)**		
White	43 (83%)	41 (79%)
Black or African-American	5 (0.1%)	5 (0.1)
Asian	1 (0.02%)	0
American Indian/Alaskan Native	0	1 (0.02%)
More than one race	0	2 (0.04%)
Other	3 (0.06%)	3 (0.06%)
**Self reported Hispanic N(% of total)**		
Yes	2 (0.04%)	4 (0.08%)
No	48 (0.92%)	46 (0.88%)
No answer	2 (0.04%)	2 (0.04%)
**Baseline Pain N(group mean) +/- SD**	50(29.5) +/- 8.5	45 (29.8) +/-8.9
**Baseline Sf-36(P) N(group mean) +/- SD**	51 (67.7) +/-24.6	49 (66.4)+/- 24.7

### Outcomes

Of the 104 subjects who underwent randomization 103 completed at least one measurement timepoint, yielding 99.0% of data for analysis. However, 8 subjects are missing baseline pain data, yielding 95/104 = 91.3% in the analysis set. General Estimating Equations were used to compare the group 1 vs. group 2 acupuncture subjects’ experienced differences in the SF-36P, and McGill Pain scale.

**Fig 2:**
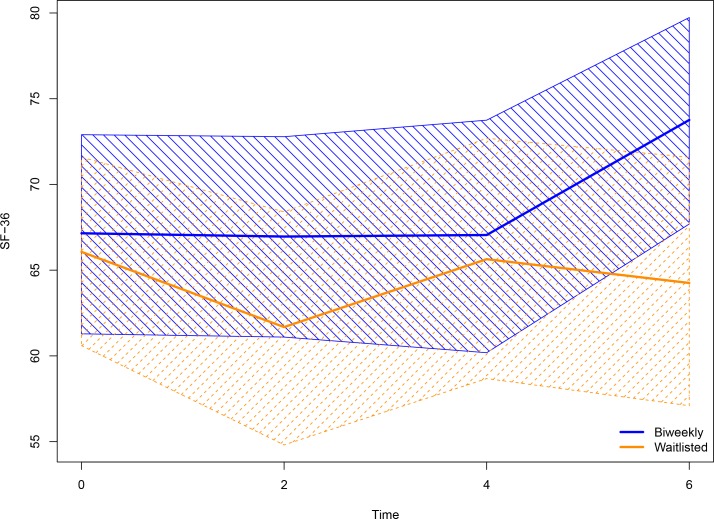
Summary of model-based simulations^xxxiii^ of changes in mean SF-36P at 4 measurement timepoints. Scores moving in the positive direction indicate improvement. Scores moving in the positive direction indicate improvement.

Our analysis comparing baseline symptom levels to those at 6 months showed a significant average increase of 9.4 points in the SF-36 physical component scale score (SF-36P) for the biweekly acupuncture group at month 6 in comparison to the weekly waitlisted group, adjusting for baseline pain. Within the biweekly group, scores were generally stable at months 0, 2, and 4, with a mean increase of 6.6 points in SF-36P observed at month 6. A nonlinear pattern was observed for the waitlisted group, though a lower SF-36P relative to baseline was estimated for all subsequent months. Table 2 offers mean and empirical 95% confidence limits estimated for both SF-36P and McGill from 5000 simulations of the fitted GEE models, and Fig 2 provides a graphical summary of this information for SF-36P [34]. Though overlapping confidence bands are observed at time points determined significantly different in the GEE fit, we note that the simulation-based approach, while useful for visual interpretation, relies on slightly different calculations than the GEE model itself.

**Table 2:** Mean and empirical 95% confidence limits estimated for both SF-36P and McGill from 5000 simulations of the fitted GEE models.

	Waitlist/Weekly SF-36P (95% CI)	Biweekly SF-36P (95% CI)	Waitlist/Weekly McGill (95% CI)	Biweekly McGill (95% CI)
Baseline	66.1 (60.6 to 71.6)	67.1 (61.2 to 72.9)	29.7 (27.1 to 32.2)	29.7 (27.4 to 32.0)
Month 2—Baseline	61.8 (54.8 to 68.6)	66.9 (61.1 to 72.5)	31.5 (28.9 to 34.1)	29.1 (27.3 to 31.0)
Month 4—Baseline	65.7 (58.4 to 72.8)	67.0 (60.1 to 72.8)	29.3 (26.7 to 31.9)	26.8 (24.6 to 29.1)
Month 6—Baseline	64.3 (57.1 to 71.4)	73.7 (67.7 to 79.8)	29.5 (26.8 to 32.0)	25.9 (23.5 to 29.3)

**Fig 3:**
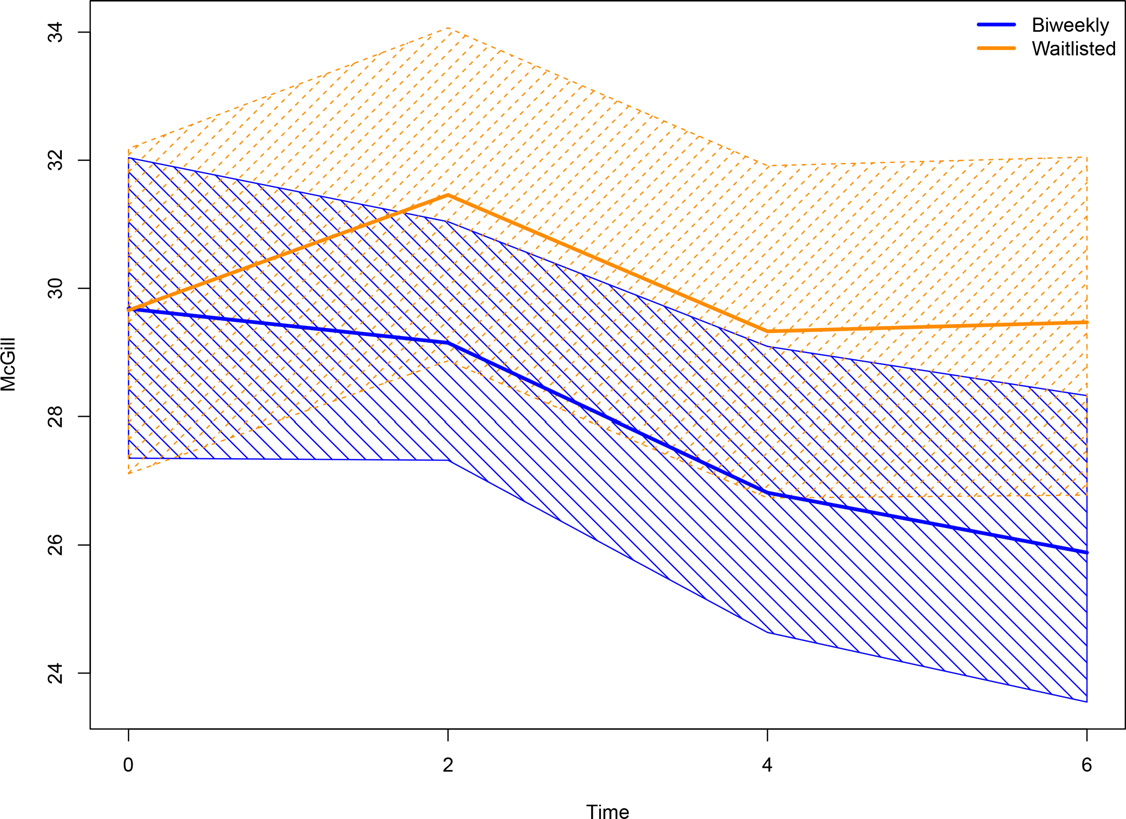
Summary of model-based simulations^xxxiii^ of changes in mean McGill Pain Scale at 4 measurement timepoints. Scores moving in the negative direction indicate improvement. Scores moving in the negative direction indicate improvement.

The secondary outcome of McGill pain index produced similar results. A decreasing trend in McGill score indicating symptom improvement was observed for the biweekly group, with a significantly lower score compared to the waitlisted group appearing at month 6. At the end of follow-up at month 6, the biweekly group was estimated to experience a reduction of approximately 3.8 points on the McGill scale compared to the weekly waitlisted group. Fig 3 illustrates the estimated value and 95% confidence intervals. Tables 3 and 4 offer estimates and accompanying statistical values from the GEE modeling of the two outcomes.

**Table 3:** Estimates and accompanying statistical values from the GEE modeling of the 6-month outcome SF-36P.

SF-36	Estimate	Std Err	95% CI	Wald X2	p-value
Intercept	106.91	6.96	(93.27, 120.56)	235.90	<0.001
Biweekly	0.98	4.07	(-7.00, 8.97)	0.06	0.81
Month 2	-4.38	2.52	(-9.32, 0.56)	3.02	0.08
Month 4	-0.42	2.97	(-6.24, 5.40)	0.02	0.89
Month 6	-1.78	3.27	(-8.19, 4.62)	0.30	0.59
Baseline pain	-1.37	0.21	(-1.79, -0.95)	41.12	<0.001
(Biweekly) X (Month 2)	4.18	3.56	(-2.81, 11.16)	1.37	0.24
Biweekly * Month 4	0.33	4.14	(-7.79, 8.46)	0.01	0.94
Biweekly * Month 6	8.39	3.76	(1.03, 15.76)	4.99	0.03

**Table 4:** Estimates and accompanying statistical values from the GEE modeling of the outcome McGill Pain scale.

McGill	Estimate	Std Err	95% CI	Wald X2	p-value
Intercept	29.65	1.28	(27.15, 32.15)	539.82	<0.001
Biweekly	0.02	1.73	(-3.38, 3.42)	0.00	0.99
Month 2	1.82	1.06	(-0.26, 3.89)	2.93	0.09
Month 4	-0.31	1.41	(-3.07, 2.45)	0.05	0.82
Month 6	-0.20	1.35	(-2.84, 2.44)	0.02	0.88
Biweekly * Month 2	-2.35	1.29	(-4.88, 0.19)	3.29	0.07
Biweekly * Month 4	-2.56	1.81	(-6.11, 0.99)	2.00	0.16
Biweekly * Month 6	-3.59	1.71	(-6.94, -0.25)	4.43	0.04

Participants reported high usability of acupuncture with 96% of the veterans (averaged across both groups and over all time points) reporting confidence in recommending acupuncture to a friend or family member (or at least a 3 on a five point scale from “Very Confident” to” Not Confident”), 98% reporting that the acupuncture experience was at least pleasant (or at least a 3 on a five point scale from “Extremely Pleasant” to “Extremely Unpleasant”), 97% reporting that their relationship with their practitioner was pleasant (or at least a 3 on a five point scale from “Extremely Pleasant” to “Extremely Unpleasant”) and 96% reported that the acupuncture treatments were logical for them (or at least a 3 on a 6 point scale of “Very Logical” to “Not Logical at All”). The trial had only two adverse events: (1) subject in biweekly treatment group reported pain on needling, (2) subject in weekly treatment group reported suicidal thoughts, which study staff followed up with additional medical oversight.

## Discussion

This study supports the use of individualized acupuncture treatments for the management of GWI symptoms. Our results are in concordance with numerous other studies indicating that acupuncture is a widely available, safe, effective, and cost-effective option for the treatment of other diseases and syndromes with similar presentations to GWI [35] with high usability in veteran populations [36]. Given this research, it is likely that acupuncture treatment may be an effective, safe, low-cost treatment option for our returning military as well as civilian populations impacted by chronic multi-symptom illness and its co-morbidities.

The mechanisms of acupuncture in the treatment of GWI are unknown, which supports our choice of a low-constraint design. This naturalistic RCT includes individualized protocols, a clinically supported length and dose of treatment, and a wait list control. Data from our wait list arm (Table 2) indicates that symptoms are stable, as has been shown in published 5- and 10- year follow-ups [1,5]. The design aspect of the wait list group eventually receiving weekly acupuncture offers us data to begin to answer questions of minimal dose and satisfies ethical concerns allowing all subjects to receive treatment during the study. A sham acupuncture control arm was not used due to published indications that such sham interventions are effective and thus not appropriate controls; very high quality evidence of this is now available [37,38].

We chose a pragmatic design, and used practitioners in the community, to facilitate adherence and test the use of extant practitioners. Our positive results support the referral of GWI veterans to acupuncture treatments. Our low side effect rate mirrors that of the published literature that acupuncture is safe when provided by professionally trained practitioners [39]. Serious adverse events are extremely rare. In a systematic review of 12 prospective studies scrutinizing over a million treatments, the very low risk of serious adverse event, mostly trauma from needle puncture or infection, was estimated at 0.05 per 10,000 treatments, a risk below that of many common medical treatments [40]. Acupuncture is well tolerated, safe and effective in the management of Gulf War Syndrome. The inclusion of acupuncture in the routine management of this intractable condition is warranted.

## Limitations and Future Directions

Our results suggest that 2 months of biweekly acupuncture is not sufficient to affect the outcome of physical function (as measured by the SF-36P), but pain scores (as measured by the McGill Pain Scale) did show group improvement as early as the first follow-up (2 months). These findings underscore the need for more dosage studies to determine the most therapeutic level of treatment for different illness presentations. Currently, we are conducting secondary data analyses exploring the effectiveness of acupuncture treatment on different subtypes of GWI to help treatment providers apply the best protocols for this complex illness. The team is also categorizing the most effective treatment protocols from a Traditional Chinese Medicine point of view and matching these with different biomedical symptom presentations. Our low-constraint/non-standardized design allowed for collection of naturalistic clinical data, which may increase data validity and make such communications across medical systems more useful.

Other items related to observation window are also of interest. For example, the obvious yet non-statistically significant decrease in Sf-36P component scores which happened while the veterans were waiting for treatment could be natural history but probably is not as observations over longer periods show symptom stability over time [41]. Most likely, this symptom change is due to veterans’ frustration in having to wait for treatment; a few of the veterans mentioned this frustration along with an acknowledgment that they were informed of the necessity for a wait list design and knowledge that they would receive treatment at 2 months. We will explore other changes which occurred during the wait list in later analyses as well as the symptom changes associated with different doses of acupuncture.

Although we did find support for our 6-month hypothesis we did not achieve the sample size determined to give us 80% power to see an effect. This leaves us more vulnerable to Type II (false negative) error. Future work should include larger sample sizes to protect against this. Larger sample sizes may also more easily support the use of standardized protocols, which become possible with the implementation of effectiveness trials such as ours that gather a range of clinically relevant treatment options

This work was supported by the Office of the Assistant Secretary of Defense for Health Affairs through the Gulf War Illness Research Program under Award No. W81XWH-09-2-0064. Opinions, interpretations, conclusions and recommendations are those of the author and are not necessarily endorsed by the Department of Defense. The sponsor was not involved in the study design; in the collection, analysis or interpretation of data; in the writing of the report; or in the decision to submit the paper for publication. Full trial protocol is available by request of the principle investigator (LC).

## Supporting Information

S1 File Protocol. (DOC)

S2 File CONSORT Checklist. (DOC)

S3 File Example Treatment Protocol for Subject in Biweekly Treatment Condition. (DOCX)

S4 File Data Used in Calculations. (XLSX)
