# Supplementary material for: The Effectiveness of Individualized Acupuncture Protocols in the Treatment of Gulf War Illness: A Pragmatic Randomized Clinical Trial
Source: PLoS One. 2016 Mar 31;11(3):e0149161. doi: 10.1371/journal.pone.0149161 (PMC4816551; doi:10.1371/journal.pone.0149161)
Supplement: S1 File — (DOC) [file pone.0149161.s001.doc]

1. Protocol Title.

**The effectiveness of acupuncture in the treatment of Gulf War Illness.**

2. Phase

This is a Phase II Clinical Trial with a usual care control arm.

3. Principle Investigator/ Study Staff

PI: Lisa Conboy MA MS ScD

Faculty and Co-director of Research, New England School of Acupuncture,

150 California Street, Newton, MA 02458

Phone: (617) 558-1788 ext 145 Fax: (617) 558-1789

Collaborator: Meredith St John, M.Ac., Dipl.Ac

Faculty, New England School of Acupuncture,

150 California Street, Newton, MA 02458

Phone: (508) 650-1680 Fax: (617) 558-1799

Collaborator: Julie Dunn, PhD

Co-director of Research, New England School of Acupuncture,

150 California Street, Newton, MA 02458

Phone: (617) 558-1788 ext 140 Fax: (617) 558-1789

Medical Monitor and Screener: Marc Goldstein MD

Director, Acupuncture Pain Clinic

Worcester Veterans Outpatient Clinic

Worcester, MA 01605

Phone: (508) 856-0104

Collaborator: Efi Kokkotou MD, ScD, PhD

Director, Neuropeptide Research Unit

Beth Israel Deaconess Medical Center

330 Brookline Avenue

Boston, MA 02215

Phone: (617) 558-1788

4. Study Locations

The study PI is Lisa Conboy, MA MS ScD. She is the co-director of research at the New England School of Acupuncture, 150 California Street, Newton, Massachusetts 02458.

Lisa Conboy MA MS ScD and Julie Dunn PhD will conduct their duties at their offices at the New England School of Acupuncture, 150 California Street, Newton, Massachusetts 02458. This is also the site where Marc Goldstein will conduct the medical screening. FWA00003759.

Biomarkers analysis will take place at Dr Kokkotou’s lab in the Dana Building at the Beth Israel Deaconess Medical Center, 330 Brookline Avenue, Boston Massachusetts 02215. FWA00003245.

Data collection and treatments will take place at the practitioners’ offices; the study practitioners have not been hired yet. One treatment site will be the office of Meredith St John, M.Ac., Dipl.Ac.,  207 Union Street,  South Natick, MA  01760. As a private practitioner she does not have a FWA or DUNs number. She does have a National Provider Identifier, established by the National Plan & Provider Enumeration System ([https://nppes.cms.hhs.gov](https://email.med.harvard.edu/OWA/redir.aspx?C=3ed563c61743466ab3f175942b5bb9ff&URL=https%3A%2F%2Fnppes.cms.hhs.gov)) which is 1255520920

5. Time Required to Complete the Study.

Assuming that the award is offered by September 30, 2009, this trial will begin immediately. The trial will run 3 years with 2 years active treatment time. The first 6 months of the trial will focus on start up activities and the last 6 months will focus on data analysis and manuscript preparation. This three-year trial will run October 1, 2009 through to October 1, 2012.

6. Background.

Gulf War Illness (GWI), or chronic multisymptom illness (CMI), is a complex illness characterized by multiple symptoms, including fatigue, sleep and mood disturbances, cognitive dysfunction, and musculoskeletal pain, which are unexplained by physical and laboratory examinations. There is no standard of care treatment for this syndrome at this time. First defined by the Centers for Disease Control and Prevention (CDC) after the first Gulf War (16), it is commonly seen with a highly individualistic presentation, associated with clusters of symptoms and co-morbid medical diagnoses, including chronic fatigue syndrome, fibromyalgia, irritable bowel syndrome, arthralgia, digestive complaints, and mood-related psychiatric disorders, including depression, posttraumatic stress disorder (PTSD), and other anxiety disorders (10,37). It has been shown to be remarkably stable at 5- and 10- year follow-ups (10,64). Of the 700,000 service personnel deployed to the Persian Gulf, 100,000 veterans of the first Gulf War (Operation Desert Shield/Storm, years 1990-1991) have presented with medical complaints through clinical and registry programs (64). GWI is twice as prevalent in deployed veterans, and seen in 15% of non-deployed veterans (10, p. 71).

CMI symptoms have been studied in cohorts of veterans in the United Kingdom, Canada, and Australia. The etiology of CMI is still unknown, and hypotheses involving exposures to vaccines, medications, pesticides, chemical munitions, and inhalation of depleted uranium dust and smoke from burning oil fields, have all been investigated inconclusively. Stress plays a likely role in the etiology of other multisymptom illnesses, such as irritable bowel syndrome (IBS), multiple chemical sensitivity and chronic headache (10 p. 73). It is likely that veterans of the current war in Iraq and Afghanistan are exposed to similar deployment stressors and will benefit from an investigation of CMI and its treatment.

Effective protocols of acupuncture treatment are available for many of the symptoms associated with this syndrome, and preliminary evidence from clinical research also supports use of acupuncture. Acupuncture is commonly used in the West in the treatment of many of the symptoms of GWI including pain (11,15). Acupuncture has shown efficacy in research trials for a variety of musculoskeletal disorders (35,7,87), and as a treatment for both acute and chronic pain after amputation in military contexts (60,31). Acupuncture has been shown to be effective in the treatment of other GWI symptoms such as fatigue (90), state, trait and situational anxiety (67), and depression (2,3,51). Further, there is evidence that acupuncture may be effective in the treatment of other complex diseases such as irritable bowel syndrome (29,44), fibromylagia (26), and post traumatic stress disorder in populations experiencing trauma (39). Further there is evidence that acupuncture treatments may utilize the same mechanisms of importance to CMI; for example, stress mediation by the autonomic nervous system and the hypothalamic-pituitary-adrenal axis (10, p. 73) and melatonin secretion (80).

Preliminary evidence from clinical research demonstrates that acupuncture is helpful in treating self-reported symptoms of posttraumatic stress disorder (PTSD) in a population of trauma victims, many of which overlap with CMI, eg., fatigue, irritability, difficulty concentrating, sleep disturbances, headache and body pain, as well as co-morbid anxiety and depression. In a randomized, controlled pilot trial of acupuncture for PTSD (n=90), acupuncture and group cognitive behavior therapy each showed large treatment effects compared to a wait-list control, at end treatment and 3-month follow-up (39). The acupuncture intervention was based on a replicable, manualized protocol, previously developed by an expert panel, using individualized diagnosis and frequent treatments. Volunteers received 2 treatments per week for 12 weeks, with a minimum of 25 needles inserted on the body and retained for 25-40 minutes, with manual stimulation, and retained vaccaria seeds on 5 auricular points (76). Last, testimonials from veterans participating in a pilot project offering acupuncture treatment for post-combat stress indicate that acupuncture is well-received in this population and include reports of improved sleep, reduced nightmares, reduced anxiety and stress, and improved mental clarity (1).

While there are no published clinical trials of acupuncture treatment for GWI, the lead acupuncturist and co-investigator on our study team (MS) has over 10 years of clinical experience treating complex diseases and symptom presentations. Many of these cases present with symptom clusters similar to veterans with GWI including fatigability and mood/cognition disorders in addition to musculoskeletal illness. This experience, coupled with research results showing that acupuncture can be effective in treating similar symptoms, argues for the examination of acupuncture in the treatment of GWI.

As mentioned above, there is much published research showing the effectiveness of acupuncture in the treatment of the symptoms of GWI. However, our study team has expertise in acupuncture research and has successfully explored the effect of acupuncture in two clinical trials of CMIs, IBS, and Chronic Pelvic. Our most recent acupuncture trial published this year in British Medical Journal (43) compared two types of acupuncture treatment versus wait-list, and supports our team’s expertise in designing effective protocols for complex ailments in the context of an RCT. This study compared the effects of two types of naturalistic patient-practitioner interaction in the context of an acupuncture trial for IBS. While also a study of acupuncture, we designed the treatment arms to address the question if the context of the interaction, either an “Augmented” warm, empathic interaction or “Limited” scientific interaction, would have an effect on outcomes. At three weeks both of the treatment groups showed significant improvement over wait list, and the Augmented group had more improvement than the limited group. The main outcomes for the study were: IBS Global Improvement Scale (IBS-GIS), Adequate Relief of IBS Symptoms (IBS-AR), IBS Symptom Severity Score (IBS-SSS) and IBS Quality of Life (IBS-QOL). The details of the design are summarized by Conboy et al. (21). At 3-weeks, the mean IBS-GIS scores (+/- standard deviation) were consistent with our hypotheses: augmented 5.0 (+/- 1.3) > limited 4.3 (+/- 1.4) > waitlist 3.8 (+/- 1.0) (p<0.0001 for trend). The proportion of patients reporting adequate relief on IBS-AR showed a similar pattern: augmented (62%) > limited (44%) > waitlist (28%) (p<0.0001). The same response trend existed in IBS-SSS (p<0.0001) and IBS-QOL (p=0.0001). All pair-wise comparisons between augmented and limited patient-practitioner relationship were significant: IBS-GIS (p<0.001), IBS-AR (p<0.001), IBS-SSS (p=0.007), IBS-QOL (p=0.01). Results were similar at 6-week follow-up (Kaptchuk et al 2008). Figure 1 summarizes the study outcomes at 3 weeks.

**Fig 1. Results of IBS trial at 3 weeks.** All four measurements of symptomatology show the same trend of improvement across treatment arms. In all cases, the acupuncture treatment arms showed significant improvement over waitlist.


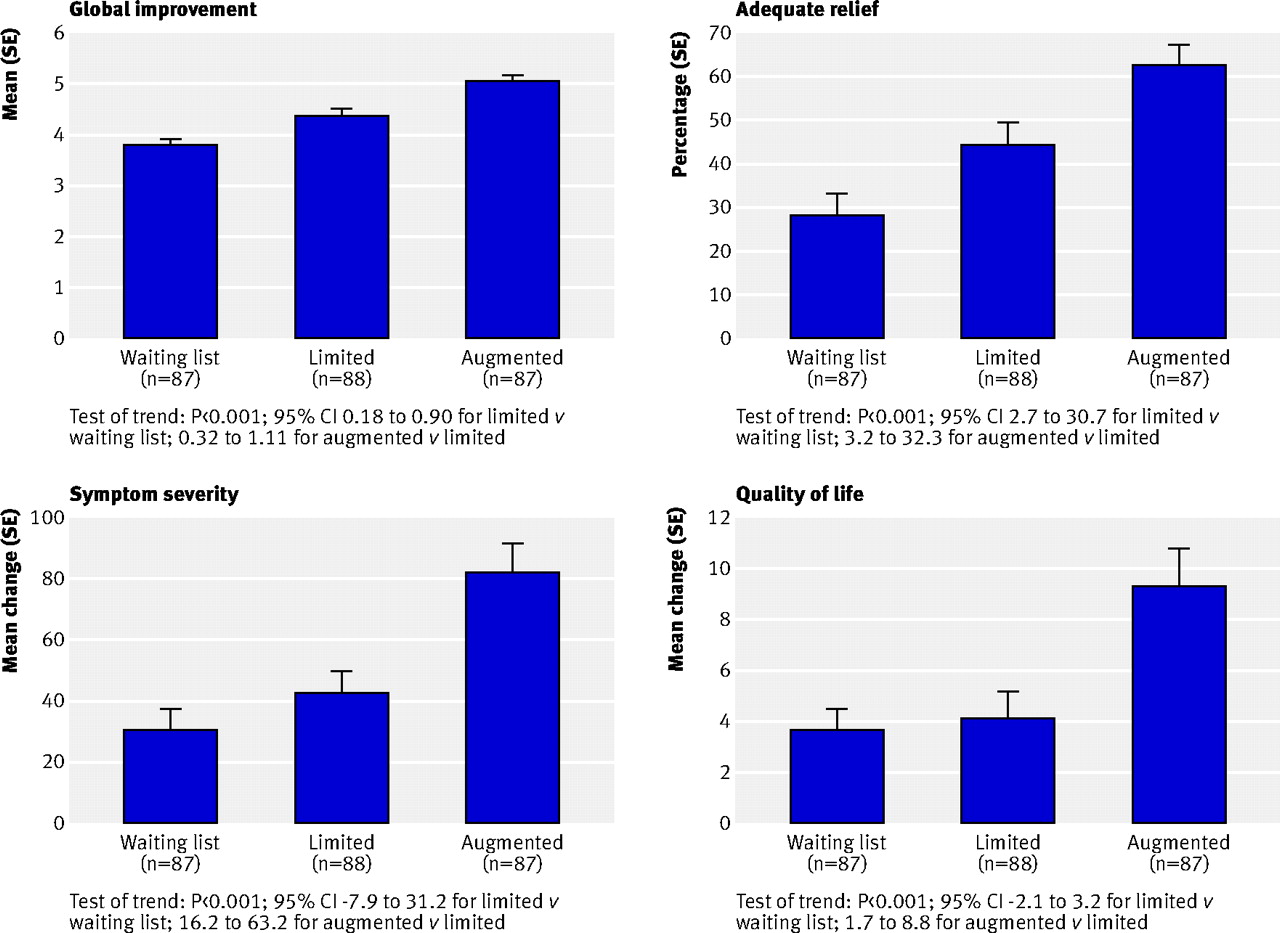


A decrease in the IBS-SSS score of 50 reliably indicates improvement in IBS symptoms (30), and our study indicates that 61% of patients in the augmented arm achieved this level of improvement at 3 weeks and this level of improvement was maintained at 6 weeks. Likewise, the changes we observed in the IBS-QOL are considered to indicate at least moderate clinical improvement in IBS symptoms (25). Finally, the percentage of patients reporting adequate relief of their IBS (62% and 61% at 3 and 6 weeks, respectively) is comparable to the responder rate in RCTs of drugs now used clinically in the treatment of IBS (13).

The IBS study was intricate and required a large sample size. Its success indicates our ability to design an implement a naturalistic acupuncture study in a sample of individuals with a CMI. We have also successfully found evidence for a positive effect of acupuncture in trials of Repetitive Strain Injury (32), and Chronic Pelvic Pain (CPP) (93). The CPP trial was a particular accomplishment because it was executed in a vulnerable population, adolescent girls, and recruitment was a particular challenge. Still we succeeded in finding support for statistically significant effects of acupuncture.

Our study team also strives to gather information to better understand the mechanisms of the disease under study and the mechanisms of acupuncture. This type of data is useful because it allows for depth and breadth in the usability of findings; we can better understand the disease in question, and our results can be directly usable by researchers in other health research specialties. For example, in the IBS trial, our study measurements also included a blood draw at the three data collection points allowing us to explore for relationships between baseline and change levels in biomarkers known or hypothesized to be related to IBS and other stress related diseases. Indeed we found interesting changes related to stress, inflammation and immune status.

For example, for our Serum biomarker analysis, fifty percent of available samples, corresponding to n=142 IBS patients, were randomly selected to be included in this preliminary analysis. Serum samples were tested using the Mesoscale arrays technology, against a highly enriched panel of 9 biomarkers included in a diagnostic test for IBS (Prometheus laboratories [www.IBSbloodtest.com](http://www.ibsbloodtest.com/).). These markers have been selected from biologically relevant pathways for IBS associated with gastrointestinal motility, brain-gut interactions, enteric neuronal activity and visceral hypersensitivity. Two examples of interesting results are given below.

**Figure 2. Serum biomarkers and responses in acupuncture treatment (phase 2, weeks 3-6)**

Using the techniques described above, across weeks 3-6 of the study, we found that women responders to phase 2 of the trial, based on pain severity as outcome, had higher levels of macrophage inflammatory protein 3-beta (MIP-3B) (p=0.0021) (Figure 2). It has been previously reported that MIP-3B positive dendritic cells were increased in the mesenteric lymph nodes of patients with Crohn’s disease (25,96). This result makes sense then given the sample.

**Figure 3: Serum biomarkers and depression: Relationship of depression at baseline and outcome.**

Our second example from the IBS study involves an examination of a particular subgroup within the sample. Seventeen percent of our IBS patients were under anti-depression treatment. We found increased baseline levels of BDNF in those patients (p=0.0058), which remained unaltered during placebo treatment (Figure 3). Experiments conducted in mice have shown that a genetic variant of BDNF (V66M) plays a key role in genetic predisposition to anxiety and depressive disorders (58). A recent meta-analysis of 11 studies comparing depressed and non-depressed studies confirmed that BDNF serum levels were lower in depressed volunteers (72). Moreover, analysis of additional 8 studies showed that BDNF levels are elevated following a course of antidepressant treatment (72), consistent with the findings of the present study. Remarkably, clinical depression was not correlated with any of our primary endpoints.

A secondary aim of the GWI project is to better define and describe this complex disease and explore potential mechanisms of healing. This can include biological changes as well as psychosocial changes. The PI of this project has worked on multiple projects to set up study designs to gather and analyze data to describe multi-level health changes. This builds on other work linking biological changes to perceived stress, other psychosocial and physical symptom changes (20,56,57). To better understand GWI, we will use validated instruments to collect information on baseline and study-related changes that may occur in the volunteers’: 1) biology, such as inflammatory cytokines associated with GWI manifestation (10,2) psychosocial attitudes and functioning such as perceived social support and improvements in social functioning, 3) symptomatology such as medical history and other symptoms. Correlations between disease factors (such as main GWI symptom) and treatment factors (such as specific acupuncture diagnosis and practitioners’ prognosis for each case) will also be considered in order to understand how acupuncture might work in the treatment of GWI. Last, toward the aim of better understanding this disease and its treatment, correlational analyses of symptom expression and treatment effectiveness by socioeconomic status, ethnicity, race, and co-morbidities will also be conducted.

For example, we examined the relationship between stress and immune function was found in the IBS data. Percent change in perceived stress from baseline was significantly associated with whether the volunteer received acupuncture or not (Pearson Correlation 2-tailed p<0.05). Percent change in perceived stress score (Pearson Correlation 2-tailed p<0.001) and perceived social support score (Pearson Correlation 2-tailed p<0.05) were significantly associated with change in IBS pain score. We next investigated whether changes in immune cells number and function, mediated by treatment arm, were associated with the clinical improvement in IBS patients. Volunteers recruited to the parental cohort during the month of April 2005 were included in the immunophenotyping study and were evaluated twice, at recruitment and at the end of the intervention (approximately 6 weeks later). Their circulating leucocytes were analyzed by flow cytometry for cell type and state of activation based on the following markers: CD3, CD4, CD8, CD25, CD56, CD 69, HLA and their various combinations. We found that the group that responded to treatment had at end point a significant 4-fold downregulation of the CD4+/CD69+ (p< 0.001) and a 3.5-fold downregulation of the CD8+/CD69+ population of activated T-lymphocytes(p<0.01, by ANOVA with Bonferroni correction)compared to non-responders (Figure 4). Although we are aware of the limitations of such a preliminary analysis (such as the various potential confounders), such results support our hypothesis of an effect of social support on the immune system and the effects of acupuncture treatment.

**Figure 4: Significant differences found between responders and nonresponders to treatment in CD4+/CD69+ and CD8+/CD69+ downregulation.**

Because GWI is a complex illness with multiple manifestations, we have chosen to design our treatment plan to offer volunteers tailored individualized treatments. Chinese Medicine, on which acupuncture is based, uses diagnostic and treatment procedures that are complex (66) and tailored to each volunteer’s specific symptoms. Although this individualized treatment ideal is often replaced in clinical research with standardized protocols for the purposes of reliability and simplicity, the complexity of the medicine is a core concept and strength of traditional acupuncture and can be maintained successfully in an RCT format (73,32,43,93) and often with better results than standardized protocols (6).

Our treatment protocol is based on a published effective protocols in similar disease (eg 76) as well as clinical experience. Due to the individualized nature of the treatments, systematic detailed records will be kept by practitioners of each treatment visit including: symptom presentation and consequent diagnosis, dose of acupuncture (measured as number of needles used and techniques applied), prognosis following that treatment. Additional clinical observations will be solicited qualitatively using an open response question format. This information is critical to best understand how acupuncture might be work and how the most effective treatment schedules can be designed. This treatment level data will also be examined for trends to better understand which specific types of treatment are most effective in the treatment of GWI.

This study has the potential to find an effective treatment for GWI that is safe and easy to administer. Multiple clinical studies have shown acupuncture to be safe, effective, and cost effective. Our study design allows us to gather specific treatment evidence applicable to the entire population of individuals with GWI. This study is also designed to gather data on the mechanisms of healing in GWI and of acupuncture; this information can be generalized toward the development of treatments of other diseases and disorders associated with military service, improving the health and quality of life of service men as well as veterans. This is an important consideration as use of Veterans Affairs mental health services have greatly increased, by Persian Gulf-era veterans, and also returning Iraq/Afghanistan veterans as well as Vietnam era veterans (71). Acupuncture has the potential to be an effective, safe, low-cost treatment option which could be of help to all of these populations.

7. Objectives/Specific Aims/Study Questions.

Objectives: To find a successful treatment for GWI, by gathering data to better understand: 1) the effectiveness of acupuncture in treating GWI; 2) the mechanisms of this disease.

Specific Aim: In a sample of veterans with GWI, evaluate the effectiveness of an individualized acupuncture treatment protocol on the volunteers’ most distressing GWI symptom.

8. Study Design.

We will utilize an unblinded randomized controlled trial design with a wait-list-control. A simple random sample will be used to obtain our sample from the accessible population. Volunteers will be randomly assigned to treatment or wait list arm using computer assignment.

Our primary outcome is quality of life. In an effort to better understand this disease and its treatment we are considering psychosocial variables (quality of life, depression, anxiety, mood), fatigue, sleep quality, and pain. All of the measurement instruments used in this trial have been used before and published on in peer reviewed scientific journals. All have shown good validity and reliability. Details of the measures are listed below in item “15. Study Procedures/Study Interventions**”.**

9. Study Population.

The study results will be generalizable to individuals with Gulf War Illness as determined by responses on the Gulf War Illness Symptom Checklist, who live within 90 miles of the Boston area and meet the inclusion/exclusion criteria as listed in “item 10 Inclusion/Exclusion Criteria” below. Participants will not be excluded due to age, race, ethnicity, or sex limitations.

Our study team has successfully recruited volunteers to acupuncture trials in populations with other complex diseases such as Irritable Bowel Syndrome (43), and Chronic Pelvic Pain (93), and in challenging populations such as a pediatric population (93). Based on our experience from other trials (18) we expect to screen four volunteers for every one that is enrolled.

As described in section “C. Advertisements, Posters, and Press Releases to Recruit Volunteers” we will be using two forms of recruitment; from the Gulf War Registry and by advertisement. The combination of these two techniques will promote timely recruitment and accrual.

10. Inclusion/Exclusion Criteria.

Inclusion:

In comparison to the original CDC definition, we chose to use a broader sampling frame. The federal definition of Gulf War Illness as used for the Gulf War Registry will be used for inclusion definition (see “Gulf War Registry Description” below). Volunteers will be invited in for screening into the study if they were: 1) deployed to the “*Gulf Theater of operations, as defined by 38 CFR 3.317, includes Iraq, Kuwait, Saudi Arabia, Bahrain, Qatar, the United Arab Emirates, Oman, the Gulf of Aden, the Gulf of Oman, the Persian Gulf, the Arabian Sea, the Red Sea, and the airspace above all of these locations”* between August 1990 and the present date, 2) they have at least 2 of the following symptoms from the 3 CDC (10) clusters of symptom that have lasted for more than 6 months. Each symptom cluster must be characterized as “mild-moderate” or “severe” with at least one symptom in each cluster required to be severe. The clusters are:

A-Fatigability

- fatigue 24 hours or more after exertion

B-Mood and Cognition

- - - - feeling depressed or
      - feeling irritable or
      - difficulty thinking or concentrating or
      - feeling worried, tense, anxious or
      - problems finding words or
      - problems getting to sleep

C-Musculoskeletal

- - - - joint pain or muscle pain

Exclusion:

Volunteers will be excluded if they are

- Currently enrolled in another clinical trial
- Have another disease that likely could account for the symptoms, as determined by our Medical Monitor
- Severe psychiatric illness (in the last 2 years psychiatric hospitalization, suicidal attempt, alcohol or substance abuse, use of antipsychotic medication) as measured by the Primary Care Evaluation of Mental Disorder (Prime MD) (81).
- Unable to complete the protocol on based on the evaluation of the Medical Monitor
- Women and/or minorities are **not** excluded

Excluded volunteers will be given a list of acupuncturists in their area should they be interested in pursuing treatment on their own.

11. Description of the Recruitment Process.

Figure 5 below is a flowchart of the Recruitment and Randomization Processes planed for the study. Our initial recruitment plan is to utilize the Gulf War Illness Database to gain contact the information of potential volunteers. The process below was offered by Erika Whittier of the VA Information Resource Center in Hines, IL as the best way to gain access to these individuals. Ms Whittier’s e-mail is attached as a letter of support.

Upon notice of study funding, the PI will send a letter to the Veterans Health Administration, Freedom of Information Act office at:

Mr. Timothy Graham

VHA FOIA Office

810 Vermont Avenue, NW (19F2)

Washington DC  20420

We will request access to the names and contact information of individuals in the Gulf War Illness Database who live within 90 miles of the Boston area. We are confident that this request will be honored as the database is currently used for research and patient contact (see Gulf War Registry Description below). Once the database is transferred to our study site it will be kept in a password protected computer and only be used for recruitment purposes. The database will be treated, and eventually destroyed, as directed by our contact at the Veterans Health Administration, Freedom of Information Act office. The following 2 paragraphs contain a description Gulf War Registry as it appears in the VHA Corporate Databases Monograph page 47 (89):

Gulf War Registry Description

***Overview***

*The Gulf War Registry (GWR) is a dataset of veterans who have participated in the Persian Gulf War, Operation Iraqi Freedom and had a GWR examination at a Department of Veterans Affairs (VA) medical facility. This centralized registry is maintained to enable VA to keep veterans informed on research findings or new compensation policies through periodic newsletters. It provides a mechanism to catalogue prominent symptoms, reproductive health, reported exposures and diagnoses, and to communicate with GW veterans. The voluntary, self-selected nature of the registry makes it valuable for health surveillance; however, it is not designed or intended to be a research tool and therefore, the results cannot be generalized to represent all GW veterans’ illnesses. The Gulf War period of conflict is between August 2, 1990, and a yet to be determined official termination date and Operation Iraqi Freedom began on March 19, 2003 through the present date. The Gulf Theater of operations, as defined by 38 CFR 3.317, includes Iraq, Kuwait, Saudi Arabia, Bahrain, Qatar, the United Arab Emirates, Oman, the Gulf of Aden, the Gulf of Oman, the Persian Gulf, the Arabian Sea, the Red Sea, and the airspace above all of these locations. The GWR is centrally housed and maintained at the CFD. Registry data is entered via Environmental Agents Service Web site into the registry database for incorporation into the registry master file. Processing occurs nightly. The primary users are the Office of Public Health and Environmental Hazards, Environmental Agents Service, and Environmental Epidemiology Service.*

***Features***

*Provides a registry for GW veterans who have had a GWR examination at a VA medical*

*facility. Provides a method to communicate with GW veterans through periodic newsletters. Provides information that may assist in generating hypotheses for future research studies. Provides on-line menu for editing and simple look-up to Austin Corporate Franchise Datacenter by the VA Central Office. Provides Veterans Health Administration (VHA) with the capability to track patient demographics, symptoms, reproductive health, reported exposures and diagnoses. Provides report generation capability* (89).

We will select volunteers from the database to be contacted using an IRB approved opt-out letter (see section “C. Advertisements, Posters, and Press Releases to Recruit Volunteers”). Because the demographics of GWI veterans are unpublished, there may be an insufficient population of GWI veterans in the Boston area. However, we will consider adding treatment sites if GWI veterans are found to be clustered in greater numbers in other parts of the New England area. One of our first study tasks upon gaining access to the GWI registry is to determine where the GWI veterans reside and what their level of interest is. This will include mailing our IRB approved recruitment letter to a sample of 100 GWI veterans in the greater Boston area, in order to gauge interest. A no-duplicates random number generator will be used to choose the names for each mailing. If we appear to have sufficient numbers the response rate can then be used to manage study flow and determine the frequency of recruitment mailings.

If recruitment using the Gulf War Registry proves insufficient after 3 months of attempts, we will utilize the advertisements material listed in section “C. Advertisements, Posters, and Press Releases to Recruit Volunteers”, which including announcements in the Boston area newspapers, and VA newsletters.

No published estimates of GWI prevalence for our catchment area are available, but we are confident that we will be able to reach our recruitment target of 120 as: 1) peer-reviewed estimates of GWI prevalence are approximately 200,000 veterans nationally (33), 2) our budget allows us to add additional treatment sites if necessary, 3) our use of multiple recruitment methods will allow us to access interested volunteers.

**Figure 5. Recruitment/Randomization Flowchart: The** **effectiveness of acupuncture in the treatment of Gulf War Illness.**

**GWI Registry**

**“Opt Out” letter**

**Opted Out (refused)**

**Advertisements**

**Initial Screening**

**(Telephone)**

**INELIGIBLE**

**Medical Screening**

**INELIGIBLE**

**Eligible Volunteers**

**Treatment**

**Waitlist**

**Follow-up**

**Follow-up**

**`**

12. Sample Size Justification.

Our study is designed to give us adequate power to detect clinically meaningful differences between treatment groups. Previous acupuncture research using our main outcome, the SF-36, in pain conditions (41,52) show a consistent standard deviation of 20 points in the Physical Component Summary score of the SF-36 for both baseline values and change scores. Sixty individuals per group (total n=120) would offer us a power of 80% to detect the difference between groups of 7 points. Using Cohen’s d estimation of effect size (20):


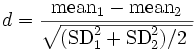


A sample size of 60 will allow us to see a moderate effect. In further support of our main outcome a 7 unit improvement has been shown to be clinically relevant (97,55,78).

Although we do not expect attrition to be large, we expect a dropout rate of no more than 10% by the 8 week endpoint. This trial is longitudinal which could increase drop-out rate. Still a veteran’s population will be less mobile and may have fewer other options for care; both of these factors should increase adherence. We will thus oversample to a target sample size of 67 volunteers per group to assure that our power to detect effects is maintained. The resulting overall sample will be 134 (one treatment group and one wait list group).

13. Description of Informed Consent Process.

The informed consent process will occur in two stages. Verbal informed consent will be administered prior to the initial telephone screening, and written informed consent will be administered in person, at the start of the screening visit.

A. Verbal Informed Consent for Telephone Screening: The first section of the Telephone Screening instrument is a script for informed consent. The Research Assistant will identify him/herself and the name of the study, determine if this is a good time for the volunteer to talk, and then give an explanation of the nature and purpose of the study. This will be similar to that provided in the written Informed Consent document but will be formatted as a series of bullet points to facilitate verbal communication between the Research Assistant and the volunteer. The volunteer will be asked if he/she has any questions. Any questions which the Research Assistant does not feel capable to answer, or for which the volunteer would like additional information, will be referred to the Study Coordinator, Principle Investigator, or Medical Advisor, as appropriate.

Next, the Research Assistant will ask the volunteer’s permission to ask him/her a series of questions to gauge eligibility for the study. The volunteer will be given an estimated time for the interview and told that some of the questions are about symptoms that he/she might experience; that some people might find some of the questions to be personal or distressing; that they can stop the interview at any time, should they choose not to continue; and that neither eligibility, participation, or non-participation in the study will in any way affect their benefits or any care that they may be receiving from the VA or elsewhere. The volunteer will be asked if they wish to continue with the screening interview, and if they say “yes”, a box indicating such will be checked on the screening form, and the date and interviewer’s initials filled in next to it. If the volunteer says “no”, they will be thanked for their time and willingness to hear about the study, the interview will be terminated, and the “No” box will be checked, dated, and initialed. At the end of the screening interview, eligible volunteers will be scheduled for an in-person screening visit at the Veteran’s Administration Hospital, Boston MA. 2 copies of the approved study consent form will be mailed to the volunteer when the appointment is scheduled. The volunteer will be encouraged to share this information with family members and medical care providers/counselors, and will be given a number to call if he/she has any additional questions prior to the screening visit.

B. Written Informed Consent process: At the beginning of the in-person screening visit, the Research Assistant will review each point of the Informed Consent document with the volunteer, in a private area. The Research Assistant will explain to the volunteer that it is possible that they may not be eligible for the study based on the results of the screening visit; however, if they are found to be ineligible, or change their mind about being in the study, they can be referred to acupuncturists in the community where they may be able to seek treatment on their own, should they wish. The volunteer will be encouraged to ask questions. If/when the staff member is assured that the volunteer understands the information in the Consent document, and is willing to proceed with secondary screening and potential enrollment in the study, the volunteer will be asked to sign 2 copies of the consent document, which will be co-signed and dated by the Research Assistant. One copy will be given to the volunteer, and the other will go into the volunteer’s chart. Should the volunteer be enrolled in the study, this document will be kept in a separate locked file from study data, along with other records containing the volunteers’ name or contact information.

Since this study is limited to United States veterans with Gulf War Syndrome, we do not expect volunteers to be minors, illiterate, or unable to speak or understand English.

14. Volunteer Screening Procedures.

Following the informed consent process volunteers will be given the check list of symptoms used to determine eligibility to the Gulf War Registry as described in “item 10 Inclusion/Exclusion”. Each volunteer will be screened by the study physician for general physical health status, physical and mental factors as described in the “Inclusion/Exclusion”, and asked to complete the Primary Care Evaluation of Mental Disorder (81).

15. Study Procedures/Study Interventions

Acupuncture intervention:

Participants will be given appointments to visit study acupuncturists for initial evaluation and treatment. During the first session, the acupuncturist will conduct an interview reviewing the volunteer’s medical history and presenting symptoms. During the physical examination, the practitioner evaluates areas of pain and aspects of diagnosis from the perspective of Traditional Chinese Medicine (TCM), including condition of the tongue, pulse, channels, and acupoints. The volunteer’s most distressing and other significant symptoms are identified and evaluated for TCM diagnosis. Each volunteer will receive an individualized treatment protocol that specifically addresses his or her unique pattern of symptoms. Individualized treatment is especially well suited for the highly variable presentation of Gulf War Illness (GWI), and pragmatic design acupuncture treatment trials have shown effectiveness for pain (91). Our study investigators have previously and with success used a technique of diagnosis and treatment called manualization (75); this technique shapes providers’ choices when best practice evidence is available allowing for improved replicability of treatments. Although symptoms of GWI have been successfully treated with acupuncture, a manualized approach would be premature. Thus we are allowing for fully individualized treatments and using accepted TCM data collection tools (included in “Section B. Surveys, Questionnaires, and Other Data Collection Instruments”) to record each treatment in detail.

Participants will receive acupuncture in private exam rooms in sessions that last about an hour. Brief interviews begin each subsequent session to review progress, prioritize symptoms, and identify any questions or concerns. After needle insertion, volunteers can expect to rest comfortably or nap. From 10 to 35 sterile, disposable needles will be inserted, with a technique characteristic of TCM known as “obtaining *de qi*,” an elicited response felt by both the patient and the acupuncturist. The needling sensation, adjusted for the comfort and safety of each patient, may be experienced as a pinch that rapidly subsides, or a sense of spreading pressure, dull ache, or warmth. Several types of needles, including from 32 to 38 gauge, and lengths from 15 mm to 50 mm, will be used on various parts of the body. Insertion depths vary by location, from subcutaneous to about 25 mm. Needles are retained 30-45 minutes. Choice of acupoints may vary during subsequent treatments to improve results.

A number of co-interventions will be used to supplement manual needling, usually 1 or 2 in each session, at the discretion of the acupuncturist. Electroacupuncture will be used for its efficacy in reducing pain and inflammation (59). Heat therapies will be used when appropriate, including heat lamp and moxibustion, a process in which a topical herbal product is heated on the needle or skin. Tui na, cupping and gua sha will be used, massage techniques in which rubbing, pressing, and scraping are applied to the body with the hands or with instruments. To prolong effects of treatment, tiny metallic balls, tacks or magnets may be applied to points after needling. The initial dose of acupuncture will be twice per week for 2 months, after which outcomes will be assessed and biological samples collected. For the following 4 months of treatment, the dose is reduced to once per week. The tapered dose of acupuncture is frequently used in clinical practice and has previously been used in research that demonstrated acupuncture efficacy for pain (9).

Volunteers in the control group will be offered 4 months of acupuncture once per week after the 2-month waiting period has elapsed. All volunteers will complete re-evaluation of outcomes instruments and biomarkers at the 6-month follow-up.

Patient charts will include complete details of each volunteer’s assessment, diagnosis, and treatment. Interview guides in use at the New England School of Acupuncture’s clinic will be used in this study: Health History Questionnaire, TCM Intake, and TCM Follow-up (included in “Section B. Surveys, Questionnaires, and Other Data Collection Instruments”). As part of standard clinical practice, the acupuncturists’ notes will include assessment of response to treatment and any adverse events. Patients’ medical records will be stored in locked offices or filing cabinets, in compliance with regulations of privacy and security mandated by the Health Insurance Portability Act of 1996 (HIPAA).

Numerous studies have shown that acupuncture is safe when provided by professionally trained practitioners. Minor adverse effects of acupuncture may include needling pain, hematoma, and slight bleeding at the needle sites, as well as transient dizziness or drowsiness. (54). Serious adverse events are extremely rare**.** In a systematic review of 12 prospective studies scrutinizing over a million treatments, the very low risk of serious adverse event, mostly trauma from needle puncture or infection, was estimated at 0.05 per 10,000 treatments, a risk below that of many common medical treatments (95).

Several hypotheses of the mechanisms of acupuncture explain its likely effects on the symptoms of GWI. Acupuncture’s effects on peripheral nerves as well as the central and autonomic nervous systems play a role in its analgesic and anti-inflammatory effects. Although the etiology of GWI is unknown, researchers suspect that the symptoms reflect a spectrum of neurologic injury (16,86). Regulation of the endocrine system, mediated by the hypothalamus-pituitary-adrenal (HPA) axis (59), suggests a likely role for acupuncture in reducing stress. While GWI is not defined as a psychiatric disorder like posttraumatic stress disorder (PTSD), stress does play a role in the etiology of other multisymptom illnesses, such as irritable bowel syndrome, multiple chemical sensitivity and chronic headache (10). Stress reduction is likely to be of significant benefit, not only to those volunteers with emotional and cognitive disturbances, but to all the volunteers.

16. Description of Protocol Drugs or Devices.

No drugs are used in this study. The devices used are described in section “15. Study Procedures/Study Interventions” above.

17. Laboratory Evaluations.

We will be collecting a small blood sample at the three data collection points and a research assistants will immediately transport the blood on ice to Dr Kokkotou’s lab. We are collecting and analyzing these data to better understand the mechanisms of GWI and changes associated with acupuncture. We are focusing on inflammatory changes, immune function, and markers of stress. For example we are interested in the relationship between acupuncture treatment, reported stress, and immune function. Multiple studies have looked at this relationship. For example recent research suggests that acupuncture modulates serum concentrations of inflammatory cytokines; for example, acupuncture reduces cytokine levels in a rat model of inflammation (79) and in asthmatic patients receiving a gentle form of Korean acupuncture (42). It is not surprising then that we have found in our IBS data set that individuals with greater decreases in reported stress had associated immunological changes as measured by a reduction in the percentage of circulating activated T-lymphocytes (CD69+). Toward the exploration of immunophenotyping, blood will continue to be collected with patient consent at the initial screen, at midpoint and at follow up. Mononuclear cells will be stained with a panel of antibodies (DAKO Cytomation) against markers of leukocyte differentiation and activation and analyzed using the Cell Quest program as described above. In parallel, serum cytokine levels (IFNγ, IL-2, IL-2R, IL-7, IL-4) will be measured by ELISA (R&D systems) and serum cortisol levels, as a surrogate biological marker for stress, by a radioimmunoassay. The evaluations at 3 different time points will enable us to identify immunological markers that are predictive of clinical response to acupuncture treatment (initial point) or are associated with it (endpoint). Initial analysis will be performed using ANOVA factorial with Bonferroni correction for multiple comparisons and ANOVA repeated measures for the overtime changes in immune status. If the results of this analysis warrant, a feature selection multivariate analysis will be performed that takes into account higher order interactions among the variables.

18. Data Analysis.

For our calculations, we will use the normal distribution assumption and explore this using a normal plot and goodness of fit test. If violations are found, non-parametric tests or transformations will be utilized. Variance equivalence will be tested using the F-test. Our protocol is designed to assure sample independence and adherence will be closely monitored. Before beginning the main statistical analyses, baseline variable levels across groups will first be considered to assess group equivalence. If the groups are not balanced on any baseline characteristics, these factors will be considered as variables in later multi-variate regressions.

The Centers for Disease Control defines Gulf War Illness (GWI) or Complex Multisymtom Illness (CMI) as the presence for at least 6 months of one or more self-reported symptoms from at least two clusters of symptoms: Cluster A (fatigability), Cluster B (Mood and Cognition), Cluster C (Musculoskeletal) (10). A single measure of severity that addresses all possible presentations of GWI does not exist. Thus we chose to use as the main outcome a general measure of health. Our primary outcome, the SF-36 (53) is a 36-item measure of global health. It is well recognized with good reliability and validity. Items address multiple aspects of physical and mental health as well as functionality. Eexperience to date from nearly 400 randomized controlled clinical trials suggests that the SF-36 is also a useful tool for evaluating the benefits of alternative treatments (88).

Comparisons between the treatment and wait-list groups will be based on improvement in SF-36 scores. We are considering the primary outcome to be the Physical Component Summary score of the SF-36. We chose to use the SF-36 in order to show general health related quality of life in this population with very diverse complaints. The Physical component subscale was chosen because it focuses on 2 of the three Centers for Disease Control GWI clusters (fatigue and musculosketal complaints). Statistically we will test mean differences between groups following 2 months of treatment using Student’s t-tests at an alpha=0.05. Analyses of secondary outcomes will include a test for mean differences between groups using student’s t-tests at an alpha=0.05.

As GWI is a complex disease with multiple symptom manifestations, we have included measures of global change as well as changes in specific symptoms of interest. These include areas suggested in the Program Announcement as well as one tailored measure described in Appendix 8) that allows volunteers to identify and report changes in their most important symptoms. Our secondary analyses will include a within-groups comparison within CDC clusters for changes baseline to endpoint (2 months of treatment). Cluster A, fatigability, will be assessed using by the Multidimensional Assessment of Fatigue (85). Cluster B, mood and cognition, will be assessed using The Profile of Mood States (62). Cluster C, musculoskeletal complaints, will be assessed using the McGill Pain Scale (55). All of these scales are well recognized in the scientific literature and have shown good reliability and validity.

Our measurements include:

Screening:

- Primary Care Evaluation of Mental Disorder (Prime MD) (81)

Data Collection:

- - The Sf-36 (53)
- Multidimensional Assessment of Fatigue (85)
- The Profile of Mood States (62)
- Pittsburg Sleep Quality Index (34)
- Measure Your Medical Outcomes Profile (40)
- Beck Anxiety Inventory (63)
- McGill Pain Scale (55)
- Carroll Depression Scale (14)
- Social support, Social Networks, and Stress (56-7)
- Medication use and Expectations for Treatment
- Blood draw to examine levels of selected markers of inflammation, stress, and immune function.

The PRIME-MD will be used as part of our medical screening procedure. It was developed by Professor Robert Spitzer and colleagues to assist general practitioners in the diagnosis of minor psychiatric disorders. The questionnaire consists of two parts, a self-report patient questionnaire, and a series of interview models for mood disorder, anxiety disorder, psychosomatic disorder, alcohol abuse and eating disorder. It shows good reliability and validity (70,81)

The SF-36 is a multi-purpose, 36 item health survey. It yields an 8-scale profile of functional health and well-being scores as well as psychometrically-based physical and mental health summary measures and a preference-based health utility index. It is a generic measure, as opposed to one that targets a specific age, disease, or treatment group. Accordingly, the SF-36 has proven useful in surveys of general and specific populations, comparing the relative burden of diseases, and in differentiating the health benefits produced by a wide range of different treatments (53).

The 16 item Multidimensional Assessment of Fatigue (MAF) scale measures four dimensions of fatigue: severity (#1-2), distress (#3), degree of interference in activities of daily living (#4-14), and timing (#15-16). Fourteen items contain numerical rating scales (#1-14) and two items have multiple-choice responses (#15-16). Respondents are asked to reflect over the past week. The MAF is a revision of the Piper Fatigue Scale, a 41-item measure of fatigue developed for research purposes and tested with oncology patients (68,85).

The Profile of Mood States (POMS) assessment has been used since1971 to measure of mood states and their fluctuations in ill and healthy populations. The POMS Standard assessment is a factor-analytically derived inventory that measures six identifiable mood or affective states. It shows good reliability, validity, and usability (61)

The Pittsburg Sleep Quality Index is a well-used and validated scale that measures various qualities of the subject’s sleep. It has shown good reliability (34).

The Measure Your Medical Outcomes Profile is a three item measure that allows participants to name their main complaints and rate the severity of them over time (40l).

The Beck Anxiety Inventory (BAI) measures the severity of anxiety in adults and adolescents. This 21 item measure can be self-administered and evaluates both physiological and cognitive symptoms of anxiety and item overlap with other self report depression inventories is minimized. Each item is descriptive of a symptom of anxiety and is rated on a scale of 0 to 3. It shows good reliability and validity (63).

The McGill Pain scale is perhaps the most recognized and utilized pain measurement device across a range of ailments. This 15 item measure asks participants to describe the quality of their pain using various different descriptors (55).

The Social Support, Social Networks, and stress measures address the quality and quantity of the volunteer’s social world. These measures have been found to correlate and predict various in stress and other health outcomes (56-7).

## ****The Carroll Depression scale is a 21 item scale which asks respondents to endorse multiple facets of depression. We have used this in our previous work and are impressed with its performance. It has good reliability and validity. (14,28).****

The study also includes a 6-month follow-up at which time the treatment group will have received 2-months of bi-weekly treatment and 4 months of weekly treatment. This treatment dose was determined from previous studies and the clinical experience of our team. The wait-list group members will have received 2 months of study attention while they served as the control component of the design. These wait list members then receive 4 months of weekly of courtesy acupuncture. Due to the unbalanced treatment schedule across the wait list and treatment groups they are not directly comparable. Yet preliminary data can be attained from each of the groups using within-groups comparisons, and student’s t-test comparisons of means. For example, changes in the wait-list scores from the main outcome timepoint (2 months) to 6 months can inform future trials considering necessary dose of treatments by highlighting the effects of weekly treatment. In this same way, we may find that while the active treatment group received more frequent sessions during the first 2 months of treatment, the 4 months of weekly treatment carry benefit as well.

19. Data Management:

Methods used for data collection:

To maintain patient confidentiality, study staff will maintain 3 databases. The first will be used for screening and patient communication only and will include necessary patient contact information. The second database will be used to maintain scores on study instruments and will not contain identifying information. A third database will be maintained to track the acupuncture treatments by volunteer identification number and will also contain no identifying information.

Data collection forms are listed in section “B. Surveys, Questionnaires, and Other Data Collection Instruments”. Our main outcome is the physical component of the Sf-36 (53).

Confidentiality:

Procedures to maintain the privacy of study volunteers and the confidentiality of data are of utmost importance. Only the Research Assistants, Medical Monitor, and Principle Investigator (if a problem is flagged) will be able to see study data with volunteer identifiers. Representatives of the USAMRMC are eligible to review study records. Further, study staff will maintain 3 databases. The first will be used for screening and patient communication only and will include necessary patient contact information. The second database will be used to maintain scores on study instruments and will not contain identifying information. A third database will be maintained to track the acupuncture treatments by volunteer identification number and will also contain no identifying information. We do not plan to collect sensitive information, but if we do become aware of such the Medical Monitor and Principle investigator will discuss the case with our Institutional Review Board, at which time an adverse event form will most likely be filed.

Disposition of data:

Our study data will be stored in electronic form with paper backup. Study data will be stored on password protect computers and locked filing cabinets at the New England School of Acupuncture. Only research staff will be able to access these files and only the Research Assistants, Medical Monitor, and Principle Investigator (if a problem is flagged) will be able to see data with patient identifiers. Questions about missing data will be referred back to the Research Assistants. Electronic data files will be entered into existing computer units. Range and logic checks will be performed including expected record lengths, study record numbers, format of variables, number of codes per encounter, and frequencies of key variables. Records or files that do not pass these checks will be corrected or recreated. A query status file will be maintained and updated at regular intervals. Completed data files will be stored and backed up. Paper copies of completed instruments will be filed by the participant’s study ID number in locked files. Strict confidentiality will be maintained. SAS programming will be used to verify data quality and create final data sets.

Sharing study results:

We do not plan to share individual study results to volunteers. Every volunteer will be provided with a study synopsis and final aggregate results if he/she so desires. We chose this option because this study collects information in the context of an experimental design and most measures might not be deemed to have clinical utility. Still, as all of our volunteers have access to regular medical care, we want volunteers to consult with their regular medical professional if they have questions. For the one biomarker with clear clinical relevance (C-reactive protein) we will provide the result to the volunteer along with a form that they can fill out to have the information sent to their regular medical professional.

20. Risks/Benefits Assessment:

Foreseeable Risks: Our data collection procedures involve minimal risk. All outcome measures have been used in multiple clinical settings without serious incident. The total intake procedure will take up to two hours and each follow up visit will last up to 60 minutes. Loss of confidentiality is a potential risk. This risk is minimized by keeping all data in locked files or computers with carefully guarded access to prevent inadvertent release of patient identifiers. All data will be identified only by the participant’s ID number. Corresponding patient names will be kept in a separate locked file. Maintaining confidentiality minimizes many risks.

Volunteers in the treatment arm will receive acupuncture which has, in rare cases, been reported to cause complications. Adverse events most commonly associated with acupuncture are minor, and serious complications are rare (19). A systematic review of 9 studies on the safety of acupuncture found the most common adverse events to be needle pain (1-45% of patients), tiredness (2-41%), and bleeding (0.03-38%)(27). More serious events were rare: faintness or syncope occurred in 0-0.3% and pneumothorax, two times in nearly a quarter million treatments(27). As with any treatment involving needle insertion, risk of infection can be minimized by good hygiene practices and use of sterile, single-use disposable needles. The most common side effect reported was a feeling of relaxation (86%) (27). Some studies have reported a transient aggravation of symptoms (1.0-2.8%), although in 86% the symptoms subsequently improved (19). Rates of adverse events decline markedly with additional training, from 2.07 adverse events per year for practitioners with <1 year to less than one per year in those with 4-5 years (19). Our protocol specifies acupuncturists with 5 or more years experience in clinical practice.

The most serious infectious problems that have been reported are hepatitis, auricular chrondritis, and bacterial endocarditis. At least three deaths have been attributed to acupuncture: one each involving pneumothorax, endocarditis and severe asthma. Minor adverse effects are more common including small hematomas and discomfort during treatment (insertion pain, smarting, drowsiness). Lao’s (45) results were similar; he concluded that maintenance of the national standard of training would “continue to ensure the safety of the practice of acupuncture.” All of our study acupuncturists are licensed and well-trained, minimizing this already low risk.

No special precautions need to be taken by volunteers as part of the study. Our well-trained practitioners are aware of particular situations that need amended care. For example, practitioners will not use electroacupuncture on volunteers with pacemakers. Knowledge of such contraindications is a standard part of acupuncture training, certification, and will be reviewed in our training. All adverse events will be reviewed by the medical monitor. If a volunteer requires emergency care during a treatment visit, he/she will escorted to the nearest emergency room if necessary and encouraged to be contact his/her usual care provider, or discuss the procedure directly with the medical monitor. The portion of the informed consent that addresses liability and cost is below:

*If I wish additional information regarding this research and my rights as a research volunteer, or if I believe I have been harmed by this study, I may contact NESA's institutional review board, New England IRB, at 781-431-7577. I am aware that this is a research project and that unforeseen side effects may occur. I understand that the New England School of Acupuncture has no formal program for compensating patients for medical injuries arising from this research. Medical referral will be provided for injuries but payment needs to be made at the usual charge to me or to my insurer unless payment is otherwise provided for in this consent form.*

*I understand that participation in this study is voluntary and I may refuse to participate or may discontinue participation at any time without penalty, loss of benefits, or prejudice to the quality of care which I will receive. I acknowledge that no guarantees have been made to me regarding the results of the treatment involved in this study, and I consent to participate in the study and have been given a copy of this form.*

The potential benefit to volunteers is free acupuncture treatment for all volunteers. As we review in our Background section, data on acupuncture in similar illnesses suggests the possibility of significant benefit. Overall, we believe the risks of participation in this study are minimal and are outweighed by the potential benefits to patients seeking relief of their GWI symptoms.

21. Study Personnel.

**Lisa Conboy, MA MS ScD (Principal Investigator)** (Year 1-40% FTE, Year 2-40% FTE, Year 3-40% FTE). Dr Conboy has a doctorate in social epidemiology with focus on the relationship between stress and the immune system. Dr. Conboy is well-published in the area of Complementary and Alternative Medicine, an Instructor in Medicine at Harvard Medical School and part-time Research Director of the New England School of Acupuncture. She has acted as chief methodologist on two studies investigating therapies for Irritable Bowel Syndrome, as well as three additional federally funded projects exploring acupuncture. She is currently the PI of a secondary data analysis project conducting novel multilevel and complex analyses of therapeutic responses and correlates of acupuncture treatments. She will have overall responsibility for the current project including protocol refinement and implementation, interpretation and presentation of findings, and preparation of manuscripts and reports. She will work closely with the Project Coordinator, other co-investigators and consultants to ensure that task assignments are clear, problems are efficiently resolved, and the timeline is adhered to. She will assist with the protocol development and oversee acupuncturists.

**Meredith St John, Lic.Ac Ac, Acupuncture Coordinator** (Year 1-40% FTE, Year 2-40% FTE, Year 3-40% FTE). Merdith St John is a faculty member at the New England School of Acupuncture and a Licensed acupuncturist with over 12 years clinical experience treating complex symptom presentations. She has worked with Dr Conboy and Rosa Schnyer on a previously funded successful acupuncture project (NESA-Harvard Acupuncture Research Collaborative (NCCAM U19 AT002022-01)) in which she focused on practitioner training. She will primarily be responsible for designing the study protocol and training and supervising the study acupuncturists. She will also participate in the semi-annual conference calls with the study consultants.

**Rosa Schnyer, LicAc, Senior Acupuncture Coordinator** (Year 1-20% FTE, Year 2-10% FTE, Year 3-10% FTE).Rosa Schyner is an acupuncturist who has written definitive books and scholarly articles on acupuncture. She has extensive experience in designing, executing and publishing the results of Randomized Controlled Trials for acupuncture**.** She has worked with Dr Conboy on three successful NIH-funded projects, one of which also included Meredith St John. She is acting here as the Senior Acupuncture Coordinator and will lead development of the treatment protocol and practitioner training and quality control.

**Julie E. Dunn, Ph.D. Data Analysis Coordinator and Recruitment Consultant.** (Year 1-10% FTE, Year 2-10% FTE, Year 3-10% FTE). Dr. Dunn is a chronic disease epidemiologist with experience in management and conduct of clinical trials (Women’s Health Initiative Northwestern University Clinical Center), observational studies (Cognitive Change in Women ancillary study to the Women’s Health Initiative (AG018695), and community-based clinical surveys. From 2003-2006 Dr. Dunn served as IRB Co-Chair at New England Research Institutes, Watertown, MA. She is presently Research Co-Director at New England School of Acupuncture and also holds an appointment at Tufts University where she teaches epidemiologic study design at the Friedman School of Nutrition Science & Policy. During Year 1 Dr. Dunn will assist the Principal Investigator with fine-tuning and operationalizing recruitment strategy. Throughout the study Dr.Dunn will oversee data management and quality assurance. In conjunction with the Biostatistical Consultant, Dr. Dunn will prepare interim data reports and final data analyses, and assist with manuscript preparation.

**Monica Shields LicAc, Project Coordinator** (Year 1-30% FTE, Year 2-30% FTE, Year 3- 30%). The project coordinator will be responsible for all administrative aspects of the project including budget review and allocation, human subjects and coordinating report writing. She will work closely with both the PI, and with the study’s Medical Monitor. She will coordinate budget allocation, assist Dr Conboy in supervising clinical research assistants, and will attend study team meetings and semi-annual conference calls with the study consultants. She will work with Dr Conboy to guide the protocol through the overseeing IRBS, HRPO, and other regulatory processes. She will have day-to-day responsibility for supervising the research assistants in implementing all procedures having to do with volunteer recruitment, data collection and data management. Monica Shields is both a licensed acupuncturist and an experienced research coordinator, and has worked with Dr Conboy to implement 2 successful federally funded projects (Enhancing the Placebo Effect in Irritable Bowel Syndrome (NCCAM R01 AT001414-01) and NESA-Harvard Acupuncture Research Collaborative (NCCAM U19 AT002022-01).

**Research Assistants (TBN)-** (Year 1-60% FTE, Year 2-60% FTE, Year 3- 60% FTE).

Two TBN research assistants will equally split this full-time position. They will be responsible for the recruitment and follow-up of patients. The research assistants are responsible for answering telephone inquiries, scheduling study volunteer appointments. They will assist all volunteers through the process of filling out of the measurement forms, as well as take the biomarker blood draw at three points in time. We plan to follow our positive experience of hiring individuals with a nursing degree (LPN or RN) as well as research experience. In this way the RAs will be able to offer the best care to the volunteers, as well as manage the blood draw and transportation of the blood to Dr Kokkotou’s lab. They will participate in regular meetings together with the Project Coordinator, the Principle Investigator, and other members of the study staff.

**Dr. Efi Kokkotou,** **MD, ScD, PhD, Biomarker Coordinator** (Year 1-10% FTE, Year 2-10% FTE, Year 3-10% FTE). Dr Kokkotou is an Assistant Professor of Medicine at Harvard Medical School and the Director of the Neuropeptide Research Unit at the Beth Israel Deaconess Medical Center, Division of Gastroenterology. Dr Kokkotou is a Physician-Scientist with training both in Medicine and Immunology (PhD) and more than 10 years experience in biomedical research. Dr Kokkotou has a solid record of publications in immunology, genetics and neuroendocrinology. She has worked on research teams with Dr Conboy as the leader of the basic science component (immunological and neuropeptide analysis) for the past 5 years. She has guided the team to make sure it remains cognizant of the importance of the development of biomarkers and understanding biological mechanisms in Complementary and Alternative treatments and taken overall responsibility for the storage and analysis of biological samples. Dr Kokkotou will lead, coordinate and be responsible for all the biomarker analyses of this grant.

**Dr.** **Roger Davis, Statistical** **consultant** (Year 1-2.5% FTE, Year 2-2.5% FTE, Year 3-2.5% FTE). will be responsible for conducting the biostatistics aspect of the study. Dr Davis is Associate Professor of Biostatistics and Associate Professor of Medicine at Harvard Medical School and author of over 224 publications. He has worked successfully with this study team on multiple trials and on this project he will oversee the database construction, randomization procedures, and analyses.

**Dr Marc Goldstein, Medical Monitor and Screener** (Year 1-15% FTE, Year 2-15% FTE, Year 3-15% FTE). Dr Goldstein is the Director of the Acupuncture Pain Clinic at Boston Veterans Healthcare System, the Jamaica Plain Campus and a Primary Care Physician, Worcester Veterans Outpatient Clinic, Worcester, MA. He will serve as the study’s medical screener and medical monitor. His experience in a veteran’s population and patients with pain qualify him for these important roles.

No member of the study team has a conflict of interest to report.

22. Roles and Responsibilities of the Medical Monitor:

The Medical Monitor will review the treatment and measurement collection for safety. As required by the HRPO and/or our individual IRBs, the medical monitor will review all adverse events, whether or not they are serious or anticipated. He will review all unanticipated problems involving risk to volunteers, study staff, and others that are associated with the protocol and provide an unbiased written report within 10 days of the event to the HRPO and the individual IRBs. This report will include a description of the event, the outcome of the event, how it is (or is not) related to the study, and if it was anticipated. The report will also state if there is agreement between the medical monitor’s report and the details as provided by the PI. If either the PI or medical monitor believes that an event is possibly or definitely related to participation or if death is a result, the HRPO and the individual IRBs will promptly receive the report.

If a potential volunteer presents with a blood level at baseline that is out of range and it has clinical utility (for example c-reactive protein) we will inform them of this and suggest that they follow-up with their health care provider as recommended by the medical monitor. As directed by the IRB, we will inform the volunteer that we are happy to send these results to his/her primary care provider with their permission. If they want to follow-up with their doctor we will collect from them their health care provider information and we will send the provider the results.

Study Organization and Management Plan

Please find below **Figure 6:Organizational Chart** which gives an overview of the responsibilities and regular communication between different roles on the study team. The PI will oversee all operations and communicate with all coordinators and consultants. The Data analysis coordinator will work directly with the statistical consultant. The acupuncture coordinator, under the direction of the PI and the Senior acupuncture coordinator will work together to organize the treatment training and protocols. The Project Coordinator will communicate with all levels of the team and directly supervises the Research Assistants. The Biomarker Coordinator mainly communicates with the PI, but her input is welcome at team meetings. The medical monitor interfaces with the PI and Data analysis coordinator to assure volunteer safety.

Figure 7 below is a timeline of study activities from start up to final manuscript preparation.

23. Study Organization and Management Plan:

**Figure 6. Organizational Chart: The** **effectiveness of acupuncture in the treatment of Gulf War Illness.**

Principle Investigator

Lisa Conboy, Ph.D.

Project Coordinator

Acupuncture Coordinator

Sr. Acupuncture Coordinator

Medical Monitor

Biomarker Coordinator

Statistical Consultant

Data Analysis Coordinator

Research Assistant

Research Assistant

Acupuncturist

Acupuncturist

Acupuncturist

**Figure 7. Timeline: The effectiveness of acupuncture in the treatment of Gulf War Illness.**

|  | **Year 1** | | **Year 2** | | **Year 3** | |
| --- | --- | --- | --- | --- | --- | --- |
| **Study Month-🡪** | 6 | 12 | 18 | 24 | 30 | 36 |
| Prepare instruments, data bases, IRB review | X |  |  |  |  |  |
| Train research assistants, acupuncturists | X |  |  |  |  |  |
| Set up databases | X |  |  |  |  |  |
| Supervision & adherence checks | X | X | X | X |  |  |
| Subject recruitment & enrollment |  | X | X | X |  |  |
| Data collection & entry |  | X | X | X | X |  |
| Data analysis |  |  |  |  | X | X |
| Annual reports submission |  | X |  | X |  | X |
| Final manuscript writing |  |  |  |  | X | X |

24. Withdrawal from the Protocol

As stated in the informed consent document volunteers may discontinue participation at any time without a penalty or loss of benefits to which he/she was previously entitled. For example, the volunteer’s regular medical care will not be affected. The same is true if the study is terminated for reasons over which the volunteer has no control (e.g. loss of funding). All volunteers will be asked to participate in an exit interview when participation has ended. This interview gathers information about the volunteer’s study experience and is an opportunity for the volunteer to learn more about the study. A list of licensed acupuncturists that practice in the volunteer’s geographic area will be offered to the volunteer, should they want to continue with acupuncture therapy.

25. Modifications to the Protocol

All modifications to the protocol will be first submitted for review to the participating IRBs prior to implementation. Any modifications that could increase the risk to volunteers must also be submitted for approval to the HRPO prior to implementation. All other amendments will be submitted as part of the continuing review report to the HRPO for acceptance. Unanticipated protocol modifications will be addressed during the weekly study staff meetings allowing the expertise of the study staff team to be reflected in the decision to make a change. The PI will discuss the potential change with the medical monitor to assess risk to volunteers. If a change is decided upon the study coordinator will work with the PI to complete and submit the necessary paperwork.

All serious protocol deviations that may have an effect on the safety, or rights of a volunteer or the integrity of the study, will be reported to the HRPO within 10 days of the event. All protocol deviations, regardless of their severity or relatedness to the study, will be reported to the participating IRBs within 10 days of the event. The study coordinator will work with the PI to complete and submit the necessary paperwork.

26. Reporting of Serious Adverse Events and Unanticipated Problems

An adverse event is any health change (or side-effect) that happens to a volunteer while he/she is participating in the study. The PI has the primary responsibility of reporting adverse events and will work with the study coordinator to submit all paperwork. All adverse events, regardless of severity, will be reported to our participating IRBs using their standard forms. These forms need to be submitted within 10 days for non-serious events. Volunteers who report a serious adverse event will be escorted to the nearest emergency room if the event happens during a study treatment. If the event happens while the volunteer is enrolled in the study, but not actively engaging in study activities, he/she will be directed to seek medical care either through his/her regular provider or through the use of a local emergency room.

This funding mechanism requires the procedures as outlined in the application on page 41, as stated in the cited text below.

“Unanticipated problems involving risk to volunteers or others, serious adverse events related to participation in the study, and all volunteer deaths related to participation in the study should be promptly reported by phone (301-619-2165), by email (hsrrb@amedd.army.mil), or by facsimile (301-619-7803) to the US Army Medical Research and Materiel Command’s Office of Research Protections, Human Research Protections Office. A complete written report should follow the initial notification. In addition to the methods above, the complete report can be sent to the US Army Medical Research and Materiel Command, ATTN: MCMR-ZB-P, 504 Scott Street, Fort Detrick, Maryland 21702-5012.”

The medical monitor is directly involved. “The medical monitor is required to review all unanticipated problems involving risk to volunteers or others, serious adverse events, and all volunteer deaths associated with the protocol and provide an unbiased written report of the event to the USAMRMC Office of Research Protections (ORP) Human Research Protections Office (HRPO). At a minimum, the medical monitor should comment on the outcomes of the event or problem and in the case of a serious adverse event or death comment on the relationship to participation in the study. The medical monitor should also indicate whether he/she concurs with the details of the report provided by the PI. Reports for events determined by either the PI or medical monitor to be possibly or definitely related to participation and reports of events resulting in death should be promptly forwarded to the HRPO.”

27. Continuation Review and Final Report

A copy of the approved continuing review report and IRB notification from our local IRBs will be submitted to the HRPO as soon as these documents become available. A copy of the approved final study report and local IRB notification will be submitted to the HRPO as soon as these documents become available.

**B. Surveys, Questionnaires, and Other Data Collection Instruments**

**(Copies of all scales are below.)**

Baseline Measurements

Screening:

- Primary Care Evaluation of Mental Disorder (Prime MD) (81)

Data Collection:

- - The Sf-36 (53)
- Multidimensional Assessment of Fatigue (85)
- The Profile of Mood States (62)
- Pittsburg Sleep Quality Index (34)
- Measure Your Medical Outcomes Profile (40)
- Beck Anxiety Inventory (63)
- McGill Pain Scale (55)
- Carroll Depression Scale (14)
- Social support, Social Networks, and Stress (56-7)
- Medication use and Expectations for Treatment
- Blood draw to examine levels of selected markers of inflammation, stress, and immune function.

Endpoint Measurements:

- - The Sf-36 (53)
- Multidimensional Assessment of Fatigue (85)
- The Profile of Mood States (62)
- Pittsburg Sleep Quality Index (34)
- Measure Your Medical Outcomes Profile (40)
- Beck Anxiety Inventory (63)
- McGill Pain Scale (55)
- Carroll Depression Scale (14)
- Social support, Social Networks, and Stress (56-7)
- Medication use and Expectations for Treatment
- Blood draw to examine levels of selected markers of inflammation, stress, and immune function.

Follow-up Measurements:

- - The Sf-36 (53)
- Multidimensional Assessment of Fatigue (85)
- The Profile of Mood States (62)
- Pittsburg Sleep Quality Index (34)
- Measure Your Medical Outcomes Profile (40)
- Beck Anxiety Inventory (63)
- McGill Pain Scale (55)
- Carroll Depression Scale (14)
- Social support, Social Networks, and Stress (56-7)
- Medication use and Expectations for Treatment
- Blood draw to examine levels of selected markers of inflammation, stress, and immune function.
- Debriefing and brief interview about study experience

**C. Advertisements, Posters, and Press Releases to Recruit Volunteers**

Recruitment will take place using two techniques.

1) The first recruitment technique will use an opt-out letter sent to randomly selected individuals in the Gulf War Registry. We will inform registrants of the Gulf War Registry who live within a 90 mile radius of Boston using the IRB approved letter below labeled “Dear Gulf War Veteran”. If this technique fails to allow us to meet recruitment projections by 3 months we add advertisements (see point number 2 below).

Lisa Conboy, DSc

New England School of Acupuncture

150 California Street

Newton, MA 02458

Dear Gulf War Veteran,

The New England School of Acupuncture is conducting a study on the effectiveness of acupuncture in the treatment of Gulf War Illness. This study is part of a research program funded by the Department of Defense. We received your contact information from the Gulf War Registry and are offering you participation in the study at no cost to you. Participation is completely voluntary and will not affect your regular medical care. Any information we collect during the study will be kept in a confidential manner.

If you choose to participate we ask that you volunteer for a period of 6 months. You will be asked to complete paper and pencil surveys and have a small amount of blood drawn (about 2 tablespoons) at three points in time: 1) when you start in the study, 2) after 2 months, 3) after 6 months. Acupuncture treatments will take place in offices around Boston and are accessible by public transportation. You will be reimbursed $6 for your travel expenses for every study visit. Following a screening visit half of volunteers will be asked to start **immediate treatment** and half will be asked to start **treatment after two months**. If you choose to participate your chances of receiving **immediate treatment** are 50% (like the flip of a coin).

Study staff are available at the phone number below to answer any questions you may have about the study (617-XXX, XXXX). If you do not want to participate in the study please return the enclosed post-card telling us that you are not interested. If we do not hear from you in four weeks we will call you to check on your interest. If you are not interested at that time please let us know. If you are interested in finding out more about the study please call (617-XXX, XXXX). If you have any questions about the legality of this study please contact our ethics board at New England IRB (617-XXX-XXXX).

Thank you.

Lisa Conboy, ScD and the Acupuncture for Gulf War Illness Project.

(617-XXX, XXXX)

2) Advertisement to be placed in Boston area papers and Veterans newsletters.

*Dear Gulf War Veterans: The New England School of Acupuncture is looking for volunteers to participate in a study on the effectiveness of acupuncture in the treatment of Gulf War Illness. This study is part of a research program funded by the Department of Defense. In exchange for your participation you will receive free acupuncture treatments. If you are interesting in finding out more please call us at (617-XXX, XXXX).*

**D. Additional Protocol Language Requirements**

The protocol will be conducted in accordance with the protocol submitted to and approved by the USAMRMC ORP HRPO and will not be initiated until written notification of approval of the research project is issued by the USAMRMC ORP HRPO.

Accurate and complete study records will be maintained and made available to representatives of the U.S. Army Medical Research and Materiel Command as a part of their responsibility to protect human subjects in research. Research records will be stored in a confidential manner so as to protect the confidentiality of subject information.

The knowledge of any pending compliance inspection/visit by the FDA, OHRP, or other government agency concerning clinical investigation or research, the issuance of Inspection Reports, FDA Form 483, warning letters or actions taken by any Regulatory Agencies including legal or medical actions and any instances of serious or continuing noncompliance with the regulations or requirements will be reported immediately to USAMRMC ORP HRPO.
