# Supplementary material for: The Effectiveness of Individualized Acupuncture Protocols in the Treatment of Gulf War Illness: A Pragmatic Randomized Clinical Trial
Source: PLoS One. 2016 Mar 31;11(3):e0149161. doi: 10.1371/journal.pone.0149161 (PMC4816551; doi:10.1371/journal.pone.0149161)
Supplement: S3 File — (DOCX) [file pone.0149161.s003.docx]

Supporting Document S2: Example Treatment Protocol for Subject in Biweekly Treatment Condition

|  | **Main Complaint (Subjective report of subject)** | **Objective signs as reported from practitioner** | **TCM Diagnosis** | **TCM strategy** | **Acupuncture Points** |
| --- | --- | --- | --- | --- | --- |
| Initial Treatment. | 1. Lower Back Pain 2. R Hamstring Pain | Observation: Appears relaxed & cared for Tongue: Red, thin yellow coating Pulse: Deep & weak | KD Qi Xu | Tonify KD Qi & KD Yin, extinguish LV Wind, regulate & nourish LV | GB20, BL18, BL23, BL40, KD3, LV3, LV8, Xijian, local points |
| Follow Up 1 | 1. Lower Back Pain 2. R Hamstring Pain | Tongue: Red, with a peeled spot Pulse: Deep, Xu, KD down | KD Yin Xu | Tonify the KD & build Yin, Extinguish LV Wind, Regulate & nourish LV | GB20, BL18, BL23, BL25, BL40, KD3, LV3, LV8, ST35 |
| Follow Up 2 | 1. Lower Back Pain 2. R Hamstring Pain 3. Stiff Knees & Feet | Tongue: Red with yellow patches (but had coffee), peeled Pulse: Deep, KD Xu | KD Yin Xu | Tonify the KD & build Yin, Regulate & nourish LV | KD3, KD10, LV8, LV3, GV4, BL23, GB20 |
| Follow Up 3 | 1. Lower Back Pain 2. R Hamstring Pain 3. Stiff Knees & Feet | Tongue: Thin, slightly yellow tongue coat but just had coffee Pulse: Empty, KD down | KD Xu | Tonify the KD & build Yin, Regulate & nourish LV, build Qi | Local hamstring, BL8, BL23, BL40, GV4, KD3, LV3, GB20, LI4 |
| Follow Up 4 | 1. Lower Back Pain 2. R Hamstring Pain 3. Stiff Knees & Feet | Tongue: peeled, brown tongue coating (Had coffee) Pulse: Soft, slow | KD Xu | Tonify KD Yin & Yang, build Qi, regulate LV | Anmien, LU7, LI4, KD3, LV3, BL18, BL23, GV4 |
| Follow Up 5 | 1. Lower Back Pain 2. R Hamstring Pain 3. Stiff Knees & Feet | Tongue: peeled, brown tongue coating (Burning coffee) Pulse: Soft slow | KD Xu | Tonify KD Yin & Yang, build Qi, regulate LV | BL18, BL23, BL25, GV4, KD3, LV3, GB20 |
| Follow Up 6 | 1. Lower Back Pain 2. R Hamstring Pain 3. Stiff Knees & Feet | Tongue: Peeled Pulse: Soft, slow | KD Xu | Tonify KD Yin & Yang, build Qi, regulate LV | BL18, BL23, BL25, BL40, GV4, KD3, LV3, Amnien |
| Follow Up 7 | 1. Lower Back Pain 2. R Hamstring Pain 3. Stiff Knees & Feet | Tongue: normal with yellow tongue coating but just had coffee Pulse: Slow, KD down | KD Xu | Tonify KD Yin & Yang, build Qi, regulate LV | Xijian, BL40, BL23, GV4, LV3, KD3, Amnien |
| Follow Up 8 | 1. Lower Back Pain 2. R Hamstring Pain 3. Stiff Knees & Feet | Tongue: Thick white tongue coating Pulse: Deep | KD Qi Xu | Tonify KD Yin & Yang, build Qi, regulate LV | GB20, GB21, BL20, BL23, BL40, GV4, LV3, KD6, SP6, KD3 |
| Follow Up 9 | 1. Lower Back Pain 2. R Hamstring Pain 3. Stiff Knees & Feet | Tongue: Very red but eating red candy Pulse: Deep, KD down | KD Qi Xu, Wind/Cold | Expel wind & cold, Tonify KD Qi | GV4, BL23, BL40, Xijian, LV3, KD3, Amnien, LU7, LI4 |
| Follow Up 10 | 1. Lower Back Pain 2. R Hamstring Pain 3. Stiff Knees & Feet | Tongue: Peeled with thin white tongue coating Pulse: Deep, soft | KD Yin Xu | Tonify KD Qi, nourish Yin, smooth LV | LV3, KD6, BL19, BL23, GV4, BL40, Amnien, GV20 |
| Follow Up 11 | 1. Lower Back Pain 2. R Hamstring Pain 3. Stiff Knees & Feet | Tongue: White tongue coating Pulse: Deep | KD Qi Xu | Tonify KD, Build Qi, move LV, nourish Yin | GB20, GB21, BL20, BL23, BL40, Xijian, LV3, KD3, SP6 |
| Follow Up 12 | 1. Lower Back Pain 2. R Hamstring Pain 3. Stiff Knees & Feet | Tongue: Patchy coating Pulse: Slowish, 3rd weak | KD Qi Xu | Tonify KD, Build Qi, move LV, nourish Yin | BL23, BL40, Xijian, KD3, ST36, Amnien |
| Follow Up 13 | 1. Lower Back Pain 2. R Hamstring Pain 3. Stiff Knees & Feet | Tongue: Peeled edges Pulse: Xu, soft | KD Qi Xu | Tonify KD, Build Qi, move LV, nourish Yin | BL23, BL40, Xijian, ST40, KD3, LU7, Amnien |
| Follow Up 14 | 1. Lower Back Pain 2. R Hamstring Pain 3. Stiff Knees & Feet | Tongue: Pale Pulse: Soft, slow | KD Xu | Tonify KD & SP, Tonify Qi | BL20, BL23, BL40, Xijian, ST40, KD3, Amnien |
| Follow Up 15 | 1. Lower Back Pain 2. R Hamstring Pain 3. Stiff Knees & Feet | Tongue: Pale Pulse: Soft | KD Qi Xu | Tonify KD & SP, Tonify Qi | BL23, BL40, SI13, GB21, GB20, LI4, KD3, LV3, SP6 |
